# A sprayable exosome-loaded hydrogel with controlled release and multifunctional synergistic effects for diabetic wound healing

**DOI:** 10.1016/j.mtbio.2025.102159

**Published:** 2025-08-05

**Authors:** Bo Liu, Lianglong Chen, Chaoyang Huang, HuiHui Zhang, Hai Zhou, Yanqi Chen, Xiaoyang Liu, Zhenyong Xiao, Kangyan Liang, Xiangtao Xie, Yi Gao, Kun Liu, Xiangdong Qi

**Affiliations:** a Department of Plastic and Aesthetic Surgery, Zhujiang Hospital, Southern Medical University, Guangzhou, Guangdong Province, China; b Department of Burns and Plastic, The Fourth Affiliated Hospital of Guangxi Medical University, Liuzhou, 545005, China; c Department of Burns, Nanfang Hospital, Southern Medical University, Jingxi Street, Baiyun District, Guangdong, 510515, China; d Guangxi Key Laboratory of Orthopedic Biomaterials Development and Clinical Translation, The Fourth Affiliated Hospital of Guangxi Medical University, Liuzhou, 545005, China; e Guangdong Qianhui Biotechnology Co., Ltd., Guangzhou, 510285, China; f Department of Hepatobiliary Surgery II, Guangdong Engineering Technology Research Center of Artificial Organ and Tissue Engineering, Guangzhou Clinical Research and Transformation Center for Artificial Liver, Institute of Regenerative Medicine, General Surgery Center, Zhujiang Hospital, Southern Medical University, Guangzhou, Guangdong Province, China; g State Key Laboratory of Organ Failure Research, Southern Medical University, Guangzhou, China; h Experimental Education/Administration Center, National Demonstration Center for Experimental Education of Basic Medical Sciences, Key Laboratory of Functional Proteomics of Guangdong Province, Department of Cell Biology, School of Basic Medical Sciences, Southern Medical University, Guangzhou, 510515, China

**Keywords:** Hydrogel, Exosomes, Acellular dermal matrix, Borneol, Diabetic wound healing

## Abstract

Diabetic wound healing is hindered by bacterial infections, oxidative stress, impaired vascularization, and chronic inflammation. Conventional dressings, limited by static drug release and single-functionality, fail to dynamically match the varying demands of different healing stages and dressing replacement frequencies. This study developed a multifunctional sprayable hydrogel dressing (Exo@AMCN) by photocrosslinking methacrylated decellularized dermal matrix co-loaded with human umbilical cord mesenchymal stem cell-derived exosomes (hUCMSC-Exo) and β-cyclodextrin-borneol inclusion complexes (CN). The hydrogel can be sprayed onto irregularly shaped wounds, with its crosslinking density and degradation kinetics precisely modulated by adjusting the photocuring duration. This tunability enables controlled release of exosomes and borneol over 2–7 days. Experimental findings demonstrate that Exo@AMCN displays excellent biocompatibility, broad-spectrum antibacterial activity (>85 % efficacy), and robust reactive oxygen species scavenging capacity. The dressing significantly boosts cell migration, fosters angiogenesis, and prompts macrophage polarization toward anti-inflammatory phenotypes. In a diabetic wound model, Exo@AMCN reduced residual wound area to 1.07 ± 1.27 % within 14 days by modulating tissue inflammation, enhancing collagen deposition, and stimulating neovascularization. This innovative approach, combining controlled drug release with multifunctional synergy, offers a promising individualized solution for managing diabetic wounds.

## Graphical abstract

This study introduces a novel approach for the targeted delivery of therapeutic agents by developing a photocrosslinkable ADM hydrogel. As illustrated in **graph,** methacrylation-modified ADM was functionalized with photosensitive double bonds and combined with lithium phenyl-2,4,6-trimethylbenzoylphosphinate (LAP) as a photoinitiator, forming a sprayable photocrosslinkable hydrogel. By regulating exposure to 405 nm blue light for different durations (10–300 s), the crosslinking density and degradation rate of the hydrogel were controlled to meet the specific requirements for dressing changes in diabetic wound healing. This system enables the simultaneous delivery of hUCMSC-Exo and β-cyclodextrin-nature borneol complexes (CN), offering antimicrobial and ROS scavenging capabilities while promoting keratinocyte migration, endothelial angiogenesis, and macrophage polarization to modulate the wound microenvironment. Evaluation through *in vivo* experiments on full-thickness skin wounds in type I diabetic mice confirmed the hydrogel's therapeutic efficacy. The synergistic effects of its dynamic adaptability and multifunctional coordination reduced the residual wound area to 1.07 ± 1.27 % within 14 days, showing significant improvements in collagen deposition and neovascularization. These findings highlight the promising potential of this smart hydrogel system for diabetic wound management.

**Figure:**
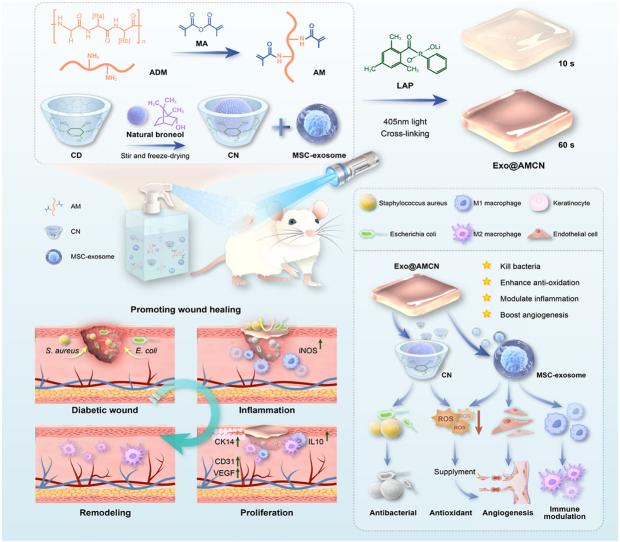

## 1 Introduction

Diabetic wounds, notably foot ulcers, pose a significant and costly complication for 15 %–25 % of individuals with diabetes globally, with 20 %–30 % of these cases necessitate limb amputation, substantially increasing overall mortality risk [1]. The impaired healing of diabetic wounds stems from a cascade of pathological events triggered by hyperglycemic and hyperlipidemic microenvironments [2]. These metabolic perturbations initiate a series of interconnected pathophysiological events, such as chronic infections resulting from pathogenic colonization, the vicious cycle of oxidative stress and inflammation, and the functional impairment of crucial reparative cells (e.g., endothelial cells, fibroblasts) [3,4]. These factors collectively trap the healing process in the inflammatory phase, preventing orderly progression to proliferation and remodeling [5,6]. Current clinical dressings face dual limitations: functional monotherapy and a fundamental mismatch between static drug release profiles and dynamic healing demands [7,8]. For instance, the exudative phase requires frequent dressing changes to remove necrotic tissue [9], whereas excessive changes during the proliferative phase disrupt nascent vascular networks and extracellular matrix (ECM) deposition [10]. Thus, developing multifunctional dressings capable of actively modulating drug release rates to align with stage-specific dressing change frequencies is critical to overcoming therapeutic bottlenecks in diabetic wound management.

Natural borneol (NB), a monoterpene bicyclic sesquiterpene compound primarily extracted from Cinnamomum camphora and other Lauraceae plants, exhibits exceptional biomembrane permeability due to its lipophilic nature [11]. Its wide-ranging antibacterial effects, ability to scavenge reactive oxygen species (ROS), and anti-inflammatory/analgesic characteristics have led to its diverse applications in fields such as ophthalmology (e.g., corneal repair), dermatology (e.g., chronic ulcers), and neurological conditions (e.g., ischemic brain injury) [12]. In wound healing, NB not only alleviates inflammation by suppressing the NF-κB pathway but also upregulates growth factors such as VEGF and FGF-2 to promote granulation tissue formation, re-epithelialization, and collagen remodeling, making it an ideal candidate for diabetic wound therapy [13]. However, its extremely low aqueous solubility severely limits formulation development and clinical efficacy [14,15]. β-Cyclodextrin (β-CD), a cyclic oligosaccharide composed of seven glucose units linked by α-1,4-glycosidic bonds, features a hydrophobic inner cavity and hydrophilic outer surface [16]. This unique structure enables host-guest inclusion complexation with NB, forming a supramolecular complex. This strategy not only significantly enhances NB's solubility but also reduces its volatility and improves thermal stability, thereby providing an ideal carrier for constructing functionalized NB delivery systems [17].

The impaired healing of diabetic wounds is attributed to three primary pathological mechanisms: persistent inflammation driven by M1 macrophage polarization, deficient angiogenesis resulting from disrupted VEGF signaling, and impaired fibroblast function in hyperglycemic microenvironments [18,19]. Conventional therapies encounter challenges in addressing these multifaceted issues simultaneously [20]. Exosomes (Exo), which are nanoscale extracellular vesicles ranging from 40 to 150 nm in diameter, offer a breakthrough by delivering functional miRNAs, cytokines, and lipid mediators [21]. This enables multidimensional modulation of the wound microenvironment, compensating for the limitations of small-molecule drugs in cellular regulation. Human umbilical cord mesenchymal stem cell-derived exosomes (hUCMSC-Exo), a prominent cell-free therapeutic approach, enhance diabetic wound healing through various mechanisms, including regulating immunity, promoting angiogenesis and stimulating cellular proliferation and differentiation [22,23]. However, the clinical translation of exosome-based therapies faces significant obstacles: native Exo are prone to degradation by proteases and demonstrate burst release in the absence of controlled delivery systems, thereby failing to maintain therapeutic levels [24]. Hydrogels are emerging as an optimal carrier for Exo due to their hydrated 3D structures, tunable physicochemical characteristics (e.g., photo-crosslinking), and resemblance to the extracellular matrix (ECM) [25]. By optimizing the pore size and degradation kinetics of hydrogels, temporal regulation of exosome release can be achieved, aligning the treatment dynamically with the sequential demands of diabetic wound healing.

Acellular Dermal Matrix (ADM) is a natural biomaterial derived from allogeneic or xenogeneic skin tissues [26]. Through physical, chemical and biological decellularization processes, epidermal and dermal cellular components are removed while retaining the structural integrity of the ECM [27]. Primarily composed of collagen, ADM exhibits excellent biocompatibility, low immunogenicity, and stable physicochemical properties. Its inherent three-dimensional fibrous network provides an optimal scaffold for cell adhesion, proliferation, and migration, supporting cell growth and differentiation to facilitate tissue regeneration and repair [28]. ECM components such as collagen interact with cell surface receptors, promoting cellular adhesion. Owing to its biocompatibility, pro-migratory, pro-proliferative, and pro-angiogenic properties, ADM has been widely applied in wound repair [29,30]. Critically, unlike synthetic polymers (e.g., PEG, PLA) or semi-synthetic derivatives (e.g., GelMA) commonly employed in photocrosslinkable hydrogels, ADM is inherently rich in native structural and functional ECM components. This authentic, multifaceted ECM composition is essential for mediating enhanced cell-ECM signaling and eliciting physiologically representative regenerative responses, thereby constituting a distinct advantage over conventional photopolymerized hydrogel systems. Nevertheless, conventional ADM encounters challenges such as limited mechanical strength and incapacity to form in situ gels, resulting in structural breakdown under dynamic wound shear forces [31]. Furthermore, commercially accessible ADM products are preformed as solid sheet-like scaffolds, lacking the ability to undergo sprayable gelation or conform to irregular wound shapes or changing tissue contraction demands [27].

**Scheme 1:**
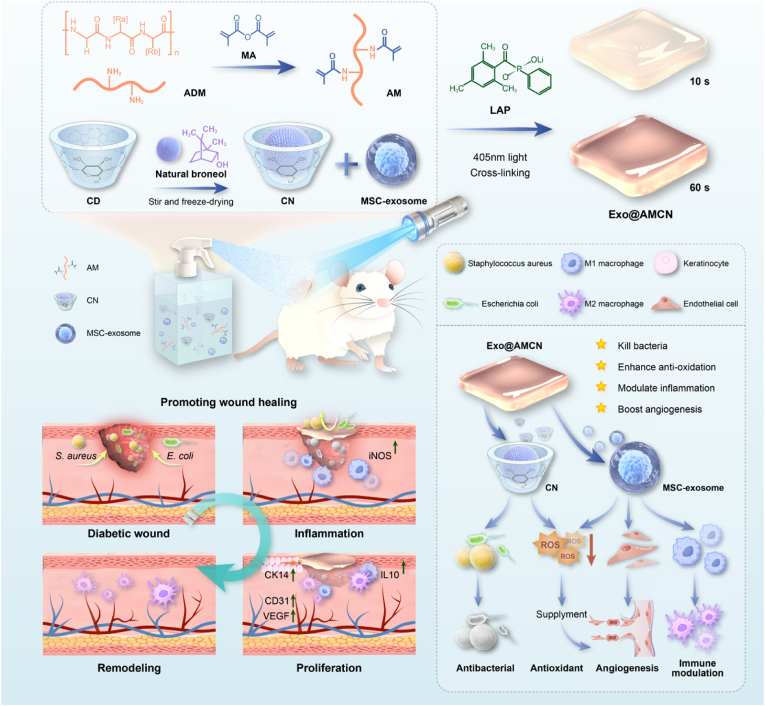
**Illustrate the synthesis and utilization of Exo@AMCN for diabetic wound healing.** (A)Preparation process of Exo@AMCN. (B)Application of Exo@AMCN in diabetic wound healing. (C)Mechanism of promoting wound healing by Exo@AMCN.

This study introduces a novel approach for the targeted delivery of therapeutic agents by developing a photocrosslinkable ADM hydrogel. As illustrated in Scheme 1, methacrylation-modified ADM was functionalized with photosensitive double bonds and combined with lithium phenyl-2,4,6-trimethylbenzoylphosphinate (LAP) as a photoinitiator, forming a sprayable photocrosslinkable hydrogel. By regulating exposure to 405 nm blue light for different durations (10–300 s), the crosslinking density and degradation rate of the hydrogel were controlled to meet the specific requirements for dressing changes in diabetic wound healing. This system enables the simultaneous delivery of hUCMSC-Exo and β-cyclodextrin-nature borneol complexes (CN), offering antimicrobial and ROS scavenging capabilities while promoting keratinocyte migration, endothelial angiogenesis, and macrophage polarization to modulate the wound microenvironment. Evaluation through *in vivo* experiments on full-thickness skin wounds in type I diabetic mice confirmed the hydrogel's therapeutic efficacy. The synergistic effects of its dynamic adaptability and multifunctional coordination reduced the residual wound area to 1.07 ± 1.27 % within 14 days, showing significant improvements in collagen deposition and neovascularization. These findings highlight the promising potential of this smart hydrogel system for diabetic wound management.

## 2 Materials and methods

### 2.1 Materials and animal care

Nature Borneol was obtained from Xinyuan Pharmaceutical (Guangzhou, China), Chitosan Bio-dressing was purchased from Zhongchuang (Hubei, China) and all chemicals and reagents were procured from Sigma-Aldrich unless otherwise stated. Human umbilical vein endothelial cells (HUVECs, Cat. No. LH-H089) were procured from Baiha Biotechnology (Shanghai, China). L929 mouse fibroblasts and RAW 264.7 macrophages were procured from the Clinical and Medical Experiment Center at Nanfang Hospital, Southern Medical University. *Escherichia coli* and *Staphylococcus aureus* strains were acquired from the Guangzhou Institute of Microbiology. Human umbilical cords were collected from healthy full-term donors at Zhujiang Hospital, Southern Medical University, following specific inclusion criteria: maternal age <35 years, negative infectious disease screening, gestational age >35 weeks, and routine prenatal care. Written informed consent was provided by all donors. The study protocol was approved by the Ethics Committees of Zhujiang Hospital (Approval No. 2019-key-015-02) and Southern Medical University.

To minimize hormonal variability, male BALB/c mice (10 weeks old, 25 ± 5 g) and Tibetan miniature pigs (male, 6 months old, ∼30 kg) were selected. The animals were procured from the Southern Medical University Animal Center (License No. SCXK [Yue] 2023-0056) and Huateng Biotechnology (Guangzhou, China) (License No. SYXK [Yue] 2024-0307). All experimental procedures were conducted in accordance with the guidelines of the Southern Hospital Animal Ethics Committee (IACUC-LAC-20240414-002). The animals were housed at 21 °C with a 12-h light/dark cycle and allowed a 7-day acclimatization period. Standard rodent/pig chow and water were provided ad libitum. Surgical anesthesia was administered by intraperitoneal injection of pentobarbital sodium at a dose of 40–50 mg/kg.

### 2.2 Exo isolation and characterization

hUCMSC-Exo were isolated through a process of differential ultracentrifugation [32]. Detailed protocols for Exo isolation and characterization are provided in the Supplementary Materials and Methods section.

### 2.3 β-cyclodextrin-nature borneol inclusion complex (CN) preparation

A 1:1 M ratio of β-CD (1135.00 mg) and NB (154.25 mg) was dissolved in 20 mL deionized water. The mixture was magnetically stirred at 400 rpm for 36 h at 25 °C, followed by freezing at −80 °C and subsequent lyophilization for 48 h, resulting in the formation of the CN complex [33].

### 2.4 Fabrication of methacrylated acellular dermal matrix (AM) hydrogels loaded with Exo and CN complexes

Porcine ADM was prepared following our previously established protocol [27]. Briefly, 0.2 g ADM powder was homogenized in 10 mL of deionized water. Acid-mediated dispersion was achieved by adding 0.1 M acetic acid to adjust pH to 3.0 under stirring (1200 rpm, 30 min). After complete disaggregation, the pH was gradually neutralized to 7.0–7.4 using 0.1 M NaOH under continuous high-shear homogenization. Under light-protected conditions, 0.3 mL methacrylic anhydride was added gradually (0.1 mL/5 min) while maintaining a pH of 8–10 with NaOH. The solution was stirred overnight, neutralized with dilute HCl, dialyzed (MWCO: 3500 kDa) for 72 h, and then lyophilized to obtain AM powder. The AM powder was reconstituted as a 2 % (w/v) solution and pipetted into cylindrical molds (10 mm diameter × 3 mm height). Photo-crosslinking was performed under 405 nm blue light (5 mW/cm^2^) for durations of 10, 60 or 300 s to produce AM_10_, AM_60_, and AM_300_ hydrogels.

To create CN-loaded AM hydrogels (AMCN), 0.1 % (w/v) LAP and 1 mg CN were homogenized in 1 mL of AM solution prior to molding and crosslinking for 10, 60 or 300 s to produce AMCN_10_, AMCN_60_, and AMCN_300_ hydrogels.

To create Exo/CN-co-loaded AM hydrogels (Exo@AMCN), 0.1 % (w/v) LAP, 1 mg CN and were 200 μg Exo were homogenized in 1 mL of AM solution prior to molding and crosslinking for 10, 60 or 300 s to produce Exo@AMCN_10_, Exo@AMCN_60_, and Exo@AMCN_300_ hydrogels.

### 2.5 Material characterization

Chemical bonds in ADM, AM_60_, and AMCN_60_ were analyzed using attenuated total reflection Fourier-transform infrared spectroscopy (ATR-FTIR; Nicolet iS50, Thermo Fisher Scientific, USA). X-ray diffraction (XRD; SmartLab SE, Rigaku, Japan) was employed to determine the phase composition and crystallinity of ADM and AM_60_.

Lyophilized ADM and hydrogels (AM_10/60/300_, Exo@AMCN_10/60/300_) were sputter-coated with gold and imaged by scanning electron microscopy (SEM; Regulus 8100, Hitachi, Japan) under high vacuum.

The viscoelastic properties of Exo@AMCN hydrogels were evaluated using a rheometer (MCR302, Anton Paar, Austria) at 37 °C with a 1 mm parallel plate gap. Frequency sweep tests (0.1–100 rad/s, 1 % strain) assessed hydrogel stability, while strain sweep tests (0.1–1000 % strain, 10 Hz frequency) determined the linear viscoelastic region and critical strain. Storage (G′) and loss (G″) moduli were recorded.

Compression and tensile tests were performed on Exo@AMCN hydrogels using a tension testing machine (DR-603 A). For compression testing, cylindrical samples (height: 8 mm, diameter: 14 mm) were compressed at a constant crosshead displacement rate of 10 mm/min until 50 % strain was achieved. For tensile testing, samples were stretched at 10 mm/min until failure. Stress-strain curves were continuously recorded throughout both tests.

hUCMSC-Exo were fluorescently labeled with PKH26. Specifically, 4 μL of PKH26 and hUCMSC-Exo were mixed in 1 mL Diluent C for 10 min, neutralized with 10 mL of 1 % bovine serum albumin, and centrifuged (100,000×*g*, 90 min). The PKH26-labeled Exo were incorporated into Exo@AMCN and visualized via confocal laser scanning microscopy (LSM 980, ZEISS, Germany).

Exo@AMCN_10_, Exo@AMCN_60_, and Exo@AMCN_300_ were individually placed into 15 mL centrifuge tubes and incubated in PBS. Following a 24-h incubation period, the hydrogels were extracted, dried the surface moisture of the hydrogels, and weigh the sample mass using an analytical balance, denoted it as M_1_. The swelling rate of the hydrogel was calculated using the following formula: Swelling rate (%) = (M_1_/M_0_) × 100 %, where M_0_ represents the initial mass of the hydrogel.

Exo@AMCN_10_, Exo@AMCN_60_, and Exo@AMCN_300_ were individually placed into 15 mL centrifuge tubes and incubate in PBS containing 2 U/mL of Type I collagenase. The tubes were then subjected to continuous agitation in a horizontal shaker at 37 °C and 100 rpm, with regular replacement of the PBS containing Type I collagenase every 2 days. The hydrogel was extracted every 8 h, the surface moisture was removed, and the sample mass was determined using an analytical balance and denoted as W_1_. The degradation rate of the hydrogel was calculated using the formula: Degradation rate (%) = [(1 - W_1_/W_0_)] × 100 %, where W_0_ represents the initial mass of the hydrogel.

### 2.6 Drug release profiling of NB and Exo

The release of NB from Exo@AMCN hydrogels was quantified using a vanillin-sulfuric acid colorimetric assay. The hydrogels were incubated in the PBS containing Type I collagenase (2 U/mL, 37 °C), with 20 μL of supernatant collected every 8 h and replaced with fresh solution. The samples were mixed with 80 μL vanillin-sulfuric acid reagent, reacted for 10 min at 25 °C, and the absorbance was measured at 470 nm (vanillin reagent as blank). Borneol concentrations were quantified against a pre-established standard curve.

Exo release was assessed using the BCA Protein Assay Kit. The hydrogels were incubated in the PBS containing Type I collagenase (2 U/mL, 37 °C), with 20 μL of supernatant collected every 8 h and replaced with fresh solution. Exosomal protein content in supernatants was determined using BCA Protein Assay Kit. The experimental design incorporated parallel controls where degradation supernatants from AMCN hydrogel were analyzed at each time point. The net exosome release was calculated as: Net Exo Release = (Protein Exo@AMCN) - (Protein AMCN). Cumulative release profiles were graphed over time.

### 2.7 Biocompatibility of hydrogels

The biocompatibility of the hydrogels was evaluated using a hemolysis assay. Fresh porcine anticoagulated whole blood was centrifuged, washed, and diluted with 0.9 % sterile physiological saline to prepare a 2 % red blood cell (RBC) suspension. AMCN, Exo, and Exo@AMCN were separately placed in centrifuge tubes, to which 1 mL of the RBC suspension was added. PBS and Triton X-100 were used as the negative and positive controls, respectively. All samples were incubated at 37 °C for 1 h, followed by centrifugation at 5000 rpm for 10 min. The supernatants were transferred to a 96-well plate, and their absorbance was measured at 540 nm using a microplate reader. The hemolysis ratio (%) was calculated using the following formula: **Hemolysis Ratio (%)** = [(ODs - ODn)/(ODp - ODn)] × 100 %, where **ODn** represents the absorbance of the PBS-treated supernatant, **ODp** represents the absorbance of the Triton X-100-treated supernatant, and **ODs** represents the absorbance of the hydrogel-treated supernatant.

The cytotoxicity of the hydrogels on L929 cells was evaluated using the CCK-8 assay. L929 cells in logarithmic growth phase were seeded into a 96-well plate at a density of 5 × 10^4^ cells per well. After cell adhesion, the original culture medium was replaced with hydrogel extracts. Following 24 h of incubation, the medium was removed and replaced with fresh medium containing 10 % CCK-8 solution, followed by incubation at 37 °C for 2 h. The supernatant was transferred to a 96-well plate, and absorbance was measured at 450 nm using a microplate reader. Cell viability (%) was calculated using the following formula: **Cell Viability (%)** = [(Cs − Cn)/(*Cc* − Cn)] × 100 %, where **Cs** represents the absorbance of the hydrogel-treated group, **Cn** represents the absorbance of the cell-free blank group, and ***Cc*** represents the absorbance of the control group.

A live/dead staining kit (Calcein-AM/PI, E-CK-A354) was used to assess the biocompatibility of the hydrogels. HUVECs in logarithmic growth phase were seeded into a 96-well plate at a density of 1 × 10^4^ cells per well. After 12 h of incubation, the culture medium was replaced with hydrogel extracts, while the control group retained DMEM medium. Following an additional 24 h of incubation, cells were washed three times with PBS. The live/dead staining solution was added and incubated in the dark for 15 min. Finally, cells were observed and imaged using an inverted fluorescence microscope (Nikon ECLIPSE Ti). Live cells fluoresced green (Calcein-AM), while dead cells fluoresced red (propidium iodide, PI).

### 2.8 Antibacterial activity of hydrogels

The antibacterial properties of the hydrogels were assessed using *Escherichia coli* (ATCC 25922) and *Staphylococcus aureus* (ATCC 6538) due to their established biosafety profiles and standardized culture conditions. Under strict aseptic conditions, 2 mL of bacterial suspension (10^7^ CFU/mL) was added to AMCN and Exo@AMCN, ensuring complete immersion of the hydrogels for optimal bacterial contact. Following incubation, the bacterial solutions were serially diluted. A 100 μL aliquot of each diluted suspension was evenly spread on agar plates, followed by 24 h of culture at 37 °C for colony counting. Agar plates inoculated with bacteria alone served as the control group, and all tests were performed in triplicate. Additionally, post-incubation bacterial solutions were fixed overnight with 2.5 % glutaraldehyde. Subsequent gradient dehydration was performed using ethanol solutions, followed by drying in a vacuum desiccator at room temperature. The ultrastructural morphology of the bacteria was then observed using SEM.

### 2.9 *In vitro* antioxidant activity of hydrogels

The antioxidant capacity of the hydrogels was evaluated using HaCaT cells. Cells in logarithmic growth phase were seeded into a 96-well plate at a density of 5 × 10^4^ cells per well. After complete adhesion, the control group was treated with 50 μM Rosup solution, the experimental group was treated with a combination of 50 μM Rosup solution and hydrogel extracts, and the blank group was supplied with serum-free basal medium. Following 12 h of incubation, cells were rinsed twice with PBS. Subsequently, 100 μL of 10 μM DCFH-DA PBS buffer was added to each well and incubated at 37 °C in the dark for 20 min. After staining, cells were washed twice with PBS. Fluorescence intensity was measured using an inverted fluorescence microscope (Nikon ECLIPSE Ti).

### 2.10 Scratch assay

To evaluate the effect of hydrogels on epithelial cell migration, HaCaT cells in the logarithmic growth phase were seeded into 12-well plates at a density of 5 × 10^5^ cells per well. After reaching confluence, two parallel, uniformly spaced scratches were created vertically on the cell monolayer using a sterile 200 μL pipette tip. Subsequently, detached cell debris was gently removed by three washes with PBS. The control group was treated with DMEM medium, while the experimental group was treated with hydrogel extracts. The plates were then incubated for 24 h, after which cell migration into the scratch area was observed and photographed under a microscope. Image J software was used for quantitative analysis of wound closure.

### 2.11 Tube formation assay

To assess the angiogenic potential of the hydrogels, Matrigel was evenly coated into 24-well confocal plates, ensuring no air bubbles, and allowed to solidify at 4 °C for 10 min, followed by 1 h in a cell culture incubator. HUVECs were seeded onto the Matrigel surface at a density of 5 × 10^4^ cells per well. The control group was cultured in high-glucose medium, while the experimental group was treated with hydrogel extracts. After 6 h of incubation in a 37 °C incubator, cells were stained using a live/dead staining kit (Calcein-AM/PI, E-CK-A354) for 20 min. Tubular structures were visualized and imaged under a fluorescence microscope. Image J software was employed to quantify tube formation parameters.

### 2.12 In vitro anti-inflammatory and angiogenic activity of hydrogels

RAW 264.7 cells and HUVECs were used to evaluate the anti-inflammatory and pro-angiogenic activity of the hydrogels *in vitro*. Cells were seeded at a density 5 × 10^4^ cells per well in standard or confocal culture dishes. After 12 h of incubation, cells were washed with PBS. The positive control group was treated with 500 ng/mL LPS solution in medium without hydrogel extracts, the experimental group was treated with 500 ng/mL LPS combined with hydrogel extracts, and the negative control group was cultured in medium alone. Following 36 h of incubation, cells were washed with PBS, fixed with 4 % paraformaldehyde for 20 min, and permeabilized with a permeabilization solution for 20 min. After blocking with 1 % bovine serum albumin (BSA) for 30 min, cells were incubated overnight at 4 °C with primary antibodies (TNF-α and IL-10 for RAW 264.7 cells, VEGF for HUVECs). FITC-conjugated goat anti-rabbit IgG (H + L) secondary antibody was then added and incubated at room temperature for 1 h. Fluorescently labeled cells were imaged using a laser confocal microscope (ZEISS LSM 980) and quantified with Image J software.

Total RNA was extracted from RAW 264.7 cells and HUVECs using the EZ-press RNA Purification Kit, followed by reverse transcription into cDNA with the RevrAid First Strand cDNA Synthesis Kit. qPCR was performed using SYBR Premix Ex Taq (TaKaRa Biotechnology, Japan) on a QuantStudio 6 Flex Real-Time PCR System (Life Technologies, China). Gene-specific primers (sequences provided in Supplementary Materials) were validated using NCBI Primer-BLAST, with GAPDH as the internal reference. The protocol included: 95 °C for 10 min (pre-denaturation), followed by 40 cycles of 95 °C for 15 s and 60 °C for 1 min, with a subsequent melt curve analysis to confirm product specificity. Relative mRNA expression levels of TNF-α, IL-10, and VEGF were calculated using the 2−ΔΔCt method. All experiments were independently repeated in triplicate.

### 2.13 In vivo wound healing experiment

Forty-five mice were randomly divided into four groups: control, CS, AMCN, Exo, and Exo@AMCN. Diabetes was induced by injecting streptozotocin (STZ, 50 mg/kg dissolved in 0.1 M citrate buffer, pH 4.5), administered every other day for three consecutive doses. On day 7 after the final injection, mice meeting diabetic criteria (random blood glucose >16.7 mM with polyuria, polydipsia, and polyphagia) were selected for subsequent experiments. Anesthesia was induced with 2 % sodium pentobarbital (40 mg/kg, intraperitoneal injection). After achieving deep anesthesia, mice were secured in a prone position on a sterile operating table. A 10-mm-diameter full-thickness skin defect was surgically created on the shaved and disinfected dorsal midline. Wounds in experimental groups were covered with respective materials and secured with 3M Tegaderm transparent film, while the control group received PBS followed by Tegaderm. Dressings were changed every 48 h, and wound healing progression was monitored on days 0, 3, 7, and 14. On day 7 post-surgery, tissues within 0.5 cm of the wound margin and major organs (heart, liver, spleen, lungs, kidneys) were collected, fixed in 4 % paraformaldehyde for 24 h, and paraffin-embedded. Histological evaluation included hematoxylin and eosin (H&E) staining, Masson's trichrome staining, immunohistochemistry (CD31 and CK14), and immunofluorescence (VEGF, iNOS and CD206) to assess re-epithelialization, inflammatory response, and angiogenesis. The local wounds and systemic responses of the mice were monitored until postoperative day 28. On postoperative day 28, venous blood samples were collected for comprehensive hematological analysis, including complete blood count (CBC) and key biochemical indicators of liver and kidney function. Concurrently, the main organs were harvested for histopathological examination. Quantitative analysis was performed using ImageJ software, with three randomly selected fields analyzed per slide for H&E staining, Masson's trichrome staining, immunohistochemistry, and immunofluorescence. Each group maintained n = 3 replicates for statistical comparisons.

### 2.14 RNA sequencing

On day 7 post-surgery, mice from the control and Exo@AMCN groups were euthanized, and wound tissues (5 mm in diameter) were aseptically excised, snap-frozen in liquid nitrogen, and stored at −80 °C. Total RNA was extracted using Trizol reagent (Invitrogen, CA, USA). RNA quantity and purity were assessed with a NanoDrop ND-1000 (NanoDrop, Wilmington, DE, USA), while RNA integrity was confirmed through analysis with Bioanalyzer 2100 (Agilent, CA, USA) and agarose gel electrophoresis. Samples meeting the following criteria (RNA concentration >50 ng/μL, RIN value > 7.0, OD260/280 ratio >1.8, and total RNA quantity >1 μg) were processed for sequencing.

After enriching mRNA with magnetic beads, fragmentation, and double-stranded cDNA synthesis, libraries were prepared through end repair, adapter ligation, and PCR amplification. Subsequently, paired-end sequencing (150 bp) was performed on the Illumina NovaSeq™ 6000 platform (LC Bio Technology Co., Hangzhou, China), yielding ≥6 Gb of raw data per sample. The raw reads were subjected to quality control using *fastp*, aligned to the reference genome (*Homo sapiens*, GRCh38) with HISAT2, and quantified via StringTie. Differentially expressed genes (DEGs) were identified with *edgeR* (screening criteria: *p* < 0.05 and |log2FC| ≥1). Finally, GO and KEGG pathway enrichment analyses were performed using DAVID.

To validate the transcriptomic findings, quantitative real-time PCR (qPCR) was performed on key genes using the same total RNA samples extracted from day 7 wound tissues. qPCR was performed using SYBR Premix Ex Taq (TaKaRa Biotechnology, Japan) on a QuantStudio 6 Flex Real-Time PCR System (Life Technologies, China). Gene-specific primers (sequences provided in Supplementary Materials) were validated using NCBI Primer-BLAST, with GAPDH as the internal reference. The protocol included: 95 °C for 10 min (pre-denaturation), followed by 40 cycles of 95 °C for 15 s and 60 °C for 1 min, with a subsequent melt curve analysis to confirm product specificity. Relative mRNA expression levels of VEGF, TNF-α, PIK3CA and AKT1 were calculated using the 2−ΔΔCt method.

### 2.15 Statistical analysis

GraphPad Prism 8 was used for data analysis. Significant differences between the groups were analyzed by means of one-way ANOVA. All the sample sizes (n) were calculated from at least triplicate samples (n ≥ 3). The data are expressed as the mean ± standard deviation and were considered statistically significantly different and reported as ∗*p,* ∗∗*p,* ∗∗∗*p* and ∗∗∗∗*p* when *p* < 0.05,*p* < 0.01,*p* < 0.001 and *p* < 0.0001 respectively.

## 3 Results and discussion

### 3.1 Preparation and characterization of hydrogels

**Fig. 1:**
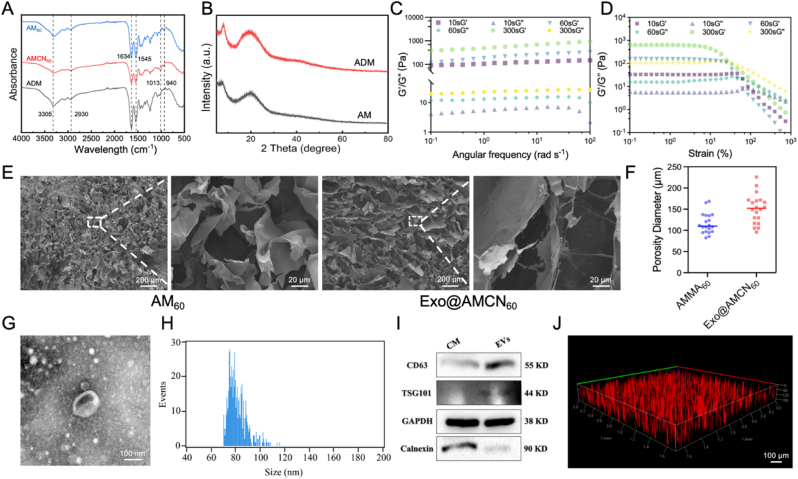
Illustrates the preparation and characterization of hydrogels. (A) FT-IR spectra of ADM, AM_60_, and AMCN_60_. (B) XRD patterns of ADM and AM. (C) G′ and G″ of hydrogels under frequency sweeps. (D) G′ and G″ of hydrogels during amplitude sweeps. (E) SEM images of AM_60_ and Exo@AMCN_60_. (F) Quantitative analysis of the pore size of hydrogels. (G) TEM image of hUCMSC-Exo. (H) The size distribution of Exo determined by NTA. (I) Western blot analysis of exosomal surface markers. (J) Confocal microscopy image of PKH27-labeled Exo loaded into Exo@AMCN.

ECM is a three-dimensional scaffold composed of proteins and polysaccharides secreted by cells [28]. Beyond providing structural support, it plays a pivotal role in regulating cellular behavior and maintaining skin homeostasis. In this study, we engineered a methacrylated decellularized dermal matrix hydrogel (AM) capable of rapid photocrosslinking. Molecular evidence of chemical modifications was confirmed via FTIR. The ADM exhibited characteristic collagen bands: amide A (3305 cm^−1^, N–H stretching), amide B (2930 cm^−1^, C–H asymmetric stretching), amide I (1634 cm^−1^, C=O stretching), and amide II (1545 cm^−1^, N–H bending/C–N stretching), which remained consistent in AM_60_ and AMCN_60_ (Fig. 1A) [34,35]. Methacrylic anhydride (MA) modification induced spectral changes: AM_60_ displayed a new peak at 940 cm^−1^ (C–H out-of-plane bending, indicative of MA double bonds) and enhanced intensity at 2930 cm^−1^, while reduced amide I/II band intensities suggested amino group involvement in MA grafting [36]. AMCN_60_ exhibited a novel peak at 1013 cm^−1^, attributed to C–O–C asymmetric stretching in the borneol ring skeleton, aligning with prior reports [37].

XRD analysis revealed the presence of characteristic peaks of native collagen at 2θ ≈ 8° (triple-helical periodicity) and 2θ ≈ 20–30° [38]. Methacrylation shifted these peaks and reduced their intensity in ADM (Fig. 1B), confirming successful MA grafting and non-covalent borneol loading into AM. Notably, borneol transitioned from poorly soluble crystalline blocks to a soluble micropowder upon forming an inclusion complex with β-CD (Fig. S1). This transformation enhanced aqueous solubility and dispersion homogeneity, optimizing borneol's functional efficacy within the hydrogel.

Rheological investigations were conducted to analyze the viscoelastic behavior of photocrosslinked hydrogels. Frequency sweep tests (Fig. 1C) demonstrated that the storage modulus (G′) of all hydrogels consistently exceeded the loss modulus (G″), confirming their elastic-dominated solid-like behavior. The parallel trend of G′ and G″ across frequencies (0.1–100 rad/s) indicated a stable topological entanglement within the Exo@AMCN hydrogel network. Prolonged photocrosslinking (300 s) significantly increased G′ from 148.01 Pa (initial) to 951.23 Pa, demonstrating enhanced crosslinking density, mechanical strength, and structural stability. Amplitude sweep tests (Fig. 1D) further revealed that the yield stress of highly crosslinked hydrogels (120.33 Pa) surpassed that of low-crosslinked counterparts (8.02 Pa), confirming superior deformation resistance. This gradient mechanical modulation strategy provides a rheological basis for designing hydrogels with tunable mechanics tailored to tissue repair requirements.

SEM images (Fig. 1E) revealed uniformly porous structures in both AM_60_ and Exo@AMCN_60_ hydrogels, with pore diameters measuring 116.33 ± 23.98 μm and 150.77 ± 33.49 μm, respectively (Fig. 1F). Notably, Exo@AMCN_60_ exhibited significantly larger pores (*p* < 0.05), possibly attribute to the competition for light absorption by exosome membrane proteins or CN, which reduced photoinitiator quantum efficiency. Consequently, this resulted in decreased crosslinking density and increased pore size.

hUCMSC-Exo were isolated and characterized for morphology, size, concentration, and surface markers. TEM imaging (Fig. 1G) confirmed their cup-shaped morphology with a diameter of approximately 100 nm. Nanoparticle tracking analysis (NTA) revealed a typical exosome size distribution (Fig. 1H). Western blotting detected exosomal markers TSG101 and CD63, while Calnexin was absent (Fig. 1I), verifying successful isolation. PKH26-labeled Exo@AMCN exhibited uniform red fluorescence under confocal microscopy (Fig. 4J), validating the incorporation of exosomes into the hydrogel. Furthermore, it is suggested that during the preparation of the hydrogel, the cell membrane structure of exosomes remains intact.

### 3.2 Applications and drug release of hydrogels

**Fig. 2:**
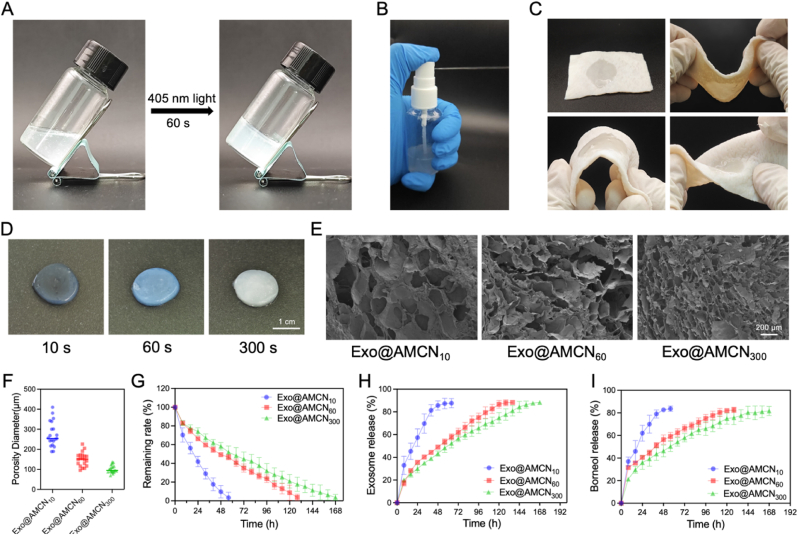
Application and drug release of hydrogels. (A) Hydrogel formed under UV irradiation. (B) Spray application of Exo@AMCN hydrogel dressing. (C) Hydrogel adapting to mechanical deformation. (D) Optical images of hydrogels with varying photocrosslinking times. (E) SEM images of hydrogels with different photocrosslinking times. (F) Quantitative analysis of hydrogels pore size. (G) Residual mass of hydrogels over time. (H) Cumulative release ratio of borneol from hydrogels. (I) Cumulative release ratio of Exo from hydrogels.

The gelation characteristics of hydrogels under 405 nm visible light irradiation are illustrated in Fig. 2A. Exo@AMCN hydrogel presents as a homogeneous liquid exhibiting excellent fluidity at ambient temperature, while demonstrating rapid photoinduced gelation upon light irradiation. This characteristic distinguishes it from conventional UV-crosslinkable hydrogels such as GelMA, which undergo spontaneous gelation at room temperature. The temperature-stable liquid state of Exo@AMCN prior to photopolymerization significantly enhances its practicality in biomedical applications. In clinical setting, sprayable hydrogel dressings with precise in situ molding capabilities are critically important for achieving complete coverage of irregular wounds. Spraying experiments confirmed the material's shape adaptability (Fig. 2B). The hydrogel precursor solution was accurately applied to target areas and solidified into an intact gel upon photocrosslinking (Fig. S2). Morphological adaptability tests further demonstrated its ability to fully fill star- and heart-shaped grooves, forming uniform solids post-crosslinking (Fig. S3). To evaluate stability in dynamic environments (e.g., joint-associated diabetic wounds), adhesion tests on porcine skin substrates showed that the hydrogel remained intact without detachment under bending and twisting (Fig. 2C). Immersion experiments in PBS revealed controlled swelling behavior, with equilibrium swelling ratios of 123.67 ± 6.03 %, 111.33 ± 2.52 %, and 104.67 ± 3.21 % for Exo@AMCN_10_, Exo@AMCN_60_, and Exo@AMCN_300_, respectively (Fig. S4), indicating robust tolerance to wound exudate. Mechanical evaluation revealed that Exo@AMCN hydrogel exhibits a compressive strength of 25.95 kPa at 50 % strain (Fig. S5). Tensile testing demonstrated a fracture elongation of 11.2 % and a tensile strength of 3.66 kPa (Fig. S6). These combined mechanical properties are crucial for ensuring the material's suitability in dynamic wound environments where both load-bearing capability and deformation tolerance are required.

Photocrosslinking-induced structural evolution was systematically investigated. Prolonged light exposure (10 s–300 s) progressively reduced hydrogel transparency (Fig. 2D), indicative of enhanced crosslinking density due to intensified network formation. To validate this hypothesis, SEM analysis revealed a striking microstructural difference: Exo@AMCN_300_ exhibited a significantly smaller pore size (101.68 ± 19.02 μm) compared to Exo@AMCN_10_ (*p* < 0.05) (Fig. 2E and F). These results conclusively demonstrate that crosslinking duration directly governs hydrogel porosity at the microscale. Consequently, the tunable microstructure of Exo@AMCN hydrogels provides a robust platform for temporally controlled drug release through precise modulation of photocrosslinking parameters.

The pore size of hydrogels significantly influences drug release kinetics by modulating molecular diffusion [39]. To evaluate the controlled release performance of Exo@AMCN hydrogels, drug release experiments were conducted under simulated physiological conditions over 7 days. Results revealed gradient degradation profiles and corresponding controlled release patterns across hydrogels with varying crosslinking densities (Fig. 2G, H, and I). At 48 h, Exo@AMCN10 achieved 90.33 ± 6.66 % degradation, accompanied by rapid release of borneol (82.67 ± 1.52 %) and Exo (85.57 ± 4.04 %). In contrast, Exo@AMCN_60_ and Exo@AMCN_300_ exhibited slower degradation (50.67 ± 2.31 % and 41.67 ± 4.04 %, respectively) and reduced release rates for borneol/Exo (56.33 ± 4.04 %/49.00 ± 1.73 % and 46.33 ± 5.13 %/43.33 ± 2.30 %, respectively), showing statistically significant differences (*p* < 0.01). By 120 h, Exo@AMCN_60_ reached 91.33 ± 8.02 % degradation with near-complete release of Exo (86.67 ± 3.51 %) and borneol (82.00 ± 2.00 %), whereas Exo@AMCN_300_ retained 21 % undegraded mass (79.00 ± 7.21 %) and released only 74.67 ± 4.72 % Exo and 75.00 ± 5.00 % borneol. Notably, Exo@AMCN_300_ achieved 96.33 ± 3.21 % degradation and sustained release of 87.67 ± 2.08 % Exo and 81.67 ± 4.04 % borneol by 168 h (7 days), demonstrating long-term release capability.

These results confirm that the hydrogel delivery system effectively delays the release of Exo and borneol, extending drug availability. By modulating photocrosslinking time, degradation cycles and release kinetics were tailored across groups: Exo@AMCN_10_ (2 days) < Exo@AMCN_60_ (5 days) < Exo@AMCN_300_ (7 days). This on-demand programmable temporal control represents a key advantage distinguishing Exo@AMCN from state-of-the-art systems: unlike the static release profile of growth factor-loaded hydrogels, our light-tunable strategy enables staged release over 2–7 days, matching characteristic wound healing phases. Furthermore, compared to “smart” responsive hydrogels, Exo@AMCN integrates both spatiotemporal precision and tunable longevity, thereby overcoming the limitation of frequent dressing changes required for dynamically evolving wounds. Consequently, our platform uniquely bridges clinical practicality with temporal controllability, surpassing current advanced hydrogel systems for diabetic wound management.

### 3.3 Biocompatibility, antibacterial activity and ROS scavenging capacity of hydrogels

**Fig. 3:**
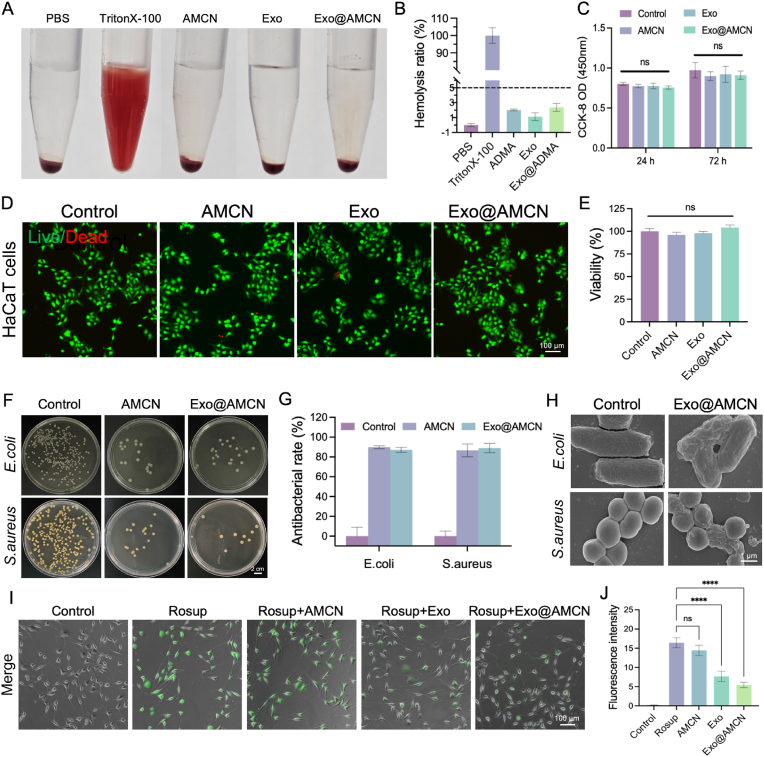
Biocompatibility, antibacterial activity, and antioxidant function of hydrogels. (A) Representative photographs of hemolysis assays for different hydrogels. (B) Hemolysis ratios of hydrogels. (C) OD values from CCK-8 assays for PBS, AMCN, Exo and Exo@AMCN. (D) Live/dead staining of HaCaT cells treated with PBS, AMCN, Exo and Exo@AMCN for 24 h. (E) Viability of HaCaTs after 24 h of treatment with PBS, AMCN, Exo and Exo@AMCN. (F) Representative images of *E. coli* and *S. aureus* colonies co-cultured with hydrogels on agar plates. (G) Antibacterial rates of AMCN and Exo@AMCN after 36 h of co-culture. (H) SEM images of *S. aureus* and *E. coli* after co-culture with Exo@AMCN. (I) DCFH-DA fluorescence in L929 cells treated with PBS, Rosup, AMCN, Exo and Exo@AMCN. (J) Relative fluorescence intensity of DCFH-DA. (∗*p* < 0.05, ∗∗*p* < 0.01, ∗∗∗*p* < 0.001, ∗∗∗∗*p* < 0.0001).

Biocompatibility and safety are prerequisites for the clinical application of biomaterials [40]. Hemolysis assays revealed that Exo, AMCN, and Exo@AMCN hydrogels exhibited hemolysis ratios below 5 %, significantly lower than the Triton X-100-positive control (*p* < 0.01) (Fig. 3A and B). CCK-8 assays confirmed the absence of cytotoxicity, as cell viability remained unaffected after co-culture with hydrogels (Fig. 3C). Additionally validation through Calcein-AM/PI dual staining demonstrated that hydrogel extracts maintained HUVEC viability above 95 % after 24 h, with intact morphology and adhesion comparable to the blank control (*p* > 0.05) (Fig. 3D and E). These results collectively confirm the excellent biocompatibility and safety of Exo@AMCN for potential biomedical applications, which is likely attributable to its naturally derived constituents.

**Fig. 4:**
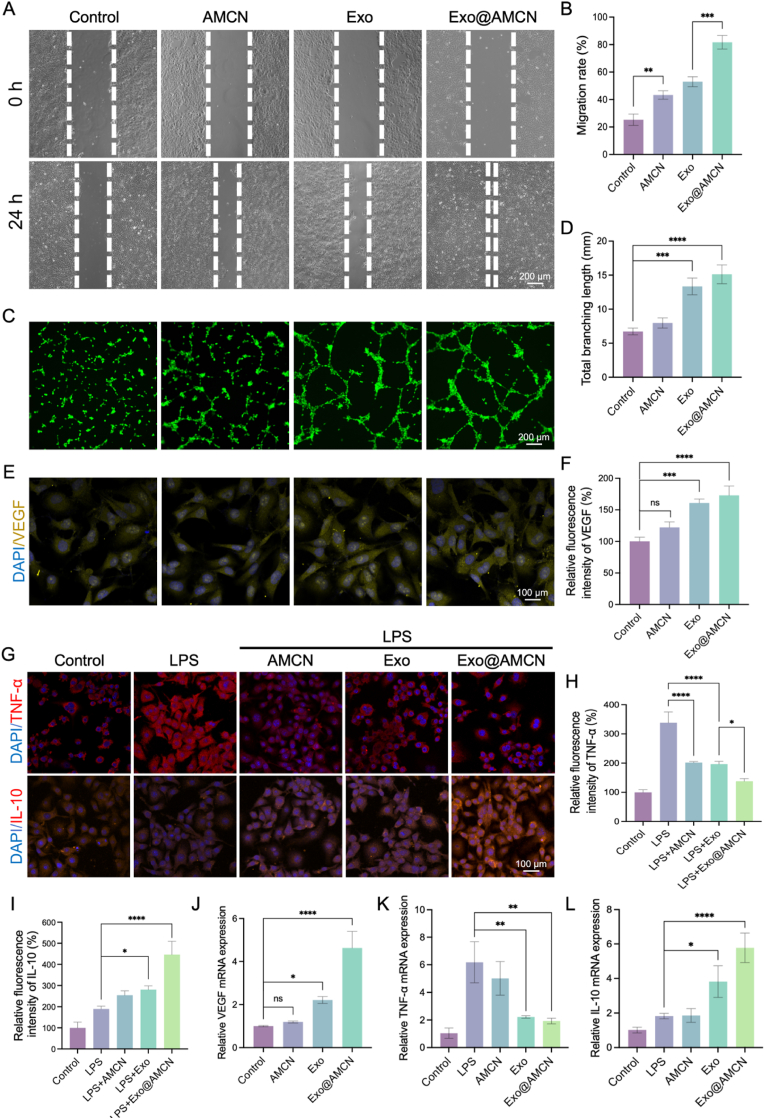
Hydrogel enhanced cell migration, angiogenesis, and inflammatory modulation. (A) Representative scratch assay images of HaCaT cells treated with hydrogel extracts for 24 h. (B) Quantification of migration rates across groups after 24 h. (C) Representative tube formation images of HUVECs cultured with hydrogel extracts. (D) Quantitative analysis of vascular network formation. (E) Immunofluorescence images of VEGF expression in HUVECs. (F) Quantitative analysis of VEGF fluorescence intensity. (G) Immunofluorescence images of TNF-α and IL-10 expression in RAW264.7 cells. (H) Quantitative analysis of TNF-α fluorescence intensity. (I) Quantitative analysis of IL-10 fluorescence intensity. (J) Relative mRNA level of VEGF. (K) Relative mRNA level of TNF-α. (L) Relative mRNA level of IL-10. (∗*p* < 0.05, ∗∗*p* < 0.01, ∗∗∗*p* < 0.001, ∗∗∗∗*p* < 0.0001).

Diabetic wounds are susceptible to polymicrobial infections, particularly by *Staphylococcus aureus* and *Escherichia coli* [41]. NB, a lipophilic compound, disrupts bacterial membrane integrity by interfering with lipid arrangements, leading to pore formation and impaired barrier function [12,13]. AMCN and Exo@AMCN hydrogels exhibited substantial antibacterial efficacy against *S. aureus* (86.67 ± 6.51 % and 89.00 ± 4.70 %) and *E. coli* (89.90 ± 1.49 % and 87.23 ± 2.48 %) (*p* < 0.001) (Fig. 3F and G). SEM revealed smooth cell walls in untreated bacteria, whereas Exo@AMCN-treated groups displayed severe membrane wrinkling (Fig. 3H), corroborating membrane disruption. These findings highlight the hydrogel's potential to suppress diabetic wound infection.

Intracellular ROS levels in L929 cells were quantified using the DCFH-DA fluorescent probe. Exo@AMCN and Exo groups showed significantly reduced green fluorescence intensity (5.45 ± 0.69 and 7.68 ± 1.36, respectively) compared to the Rosup-positive control (*p* < 0.01), indicating robust intracellular ROS scavenging (Fig. 3I). In contrast, AMCN (14.43 ± 1.34) showed no significant difference from the control (*p* > 0.05). However, *in vitro* free radical scavenging assays revealed no extracellular antioxidant activity (Fig. S5), which indicates that the hydrogels has no direct free radical scavenging ability *in vitro*. The reduction of ROS may occur through indirect mechanisms, such as regulating the activity of intracellular antioxidant enzymes or signaling pathways.

### 3.4 Pro-migratory, pro-angiogenic, and anti-inflammatory functions of hydrogels

Cell migration refers to the movement of cells in response to migratory signals or gradients of specific substances. The scratch assay, which simulates cell migration during *in vivo* wound healing, is a widely used method to evaluate cell migration and repair capabilities [42]. As shown in Fig. 4A, HaCaTs co-cultured with Exo@AMCN exhibited the fastest migration rate, achieving 81.82 ± 5.05 % wound closure after 24 h (Fig. 4B). Furthermore, both the AMCN and Exo groups demonstrated significantly increased migration distances compared to the control group (*p* < 0.05). These findings suggest that the decellularized dermal matrix and Exo independently promote cell migration, and their synergistic interaction further enhances this process, which is critical for accelerating diabetic wound healing.

Angiogenesis, a pivotal process in diabetic wound healing, provides oxygen and nutrients to damaged tissues [43]. The pro-angiogenic capacity of Exo@AMCN was assessed through a tube formation assay. As shown in Fig. 4C, Exo and Exo@AMCN groups exhibited significantly more tubular connections compared to the control, with statistically significant differences in network formation (Fig. 4D). VEGF, a specific regulator of angiogenesis, showed weak expression in normally cultured HUVECs. However, co-culture with Exo and Exo@AMCN significantly enhanced VEGF fluorescence intensity (161.00 ± 6.24 and 173.00 ± 14.73, respectively; *p* < 0.001), while no change was observed in the AMCN group (Fig. 4E and F). RT-qPCR further confirmed elevated VEGF mRNA levels in Exo and Exo@AMCN groups (*p* < 0.01) (Fig. 4J). These results indicate that Exo@AMCN upregulates VEGF expression in endothelial cells, promoting vascularization and nutrient delivery to accelerate wound repair.

Studies suggest that both Exo and borneol possess immunomodulatory properties [13,21]. The anti-inflammatory effects of Exo@AMCN were evaluated using an LPS-stimulated RAW264.7 macrophage model. As shown in Fig. 4G, control macrophages exhibited weak TNF-α expression, whereas LPS treatment induced strong TNF-α fluorescence (338.31 ± 36.82 %) and minimal IL-10 fluorescence (189.54 ± 13.07 %), indicating M1 polarization. Co-culture with AMCN, Exo, and Exo@AMCN reduced TNF-α expression (Fig. 4H) and enhanced IL-10 levels (Fig. 4I). RT-qPCR analysis confirmed these observations: LPS significantly increased TNF-α mRNA, while AMCN, Exo, and Exo@AMCN reversed this effect (*p* < 0.001), with Exo@AMCN showing the strongest suppression (Fig. 4K and L). Additionally, both Exo and Exo@AMCN groups exhibited elevated IL-10 mRNA levels compared to LPS, highlighting their anti-inflammatory activity. These results demonstrate that both Exo and borneol synergistically regulate macrophage polarization, shifting the balance from a pro-inflammatory (M1) to an anti-inflammatory (M2) phenotype.

### 3.5 In vivo diabetic wound healing

**Fig. 5:**
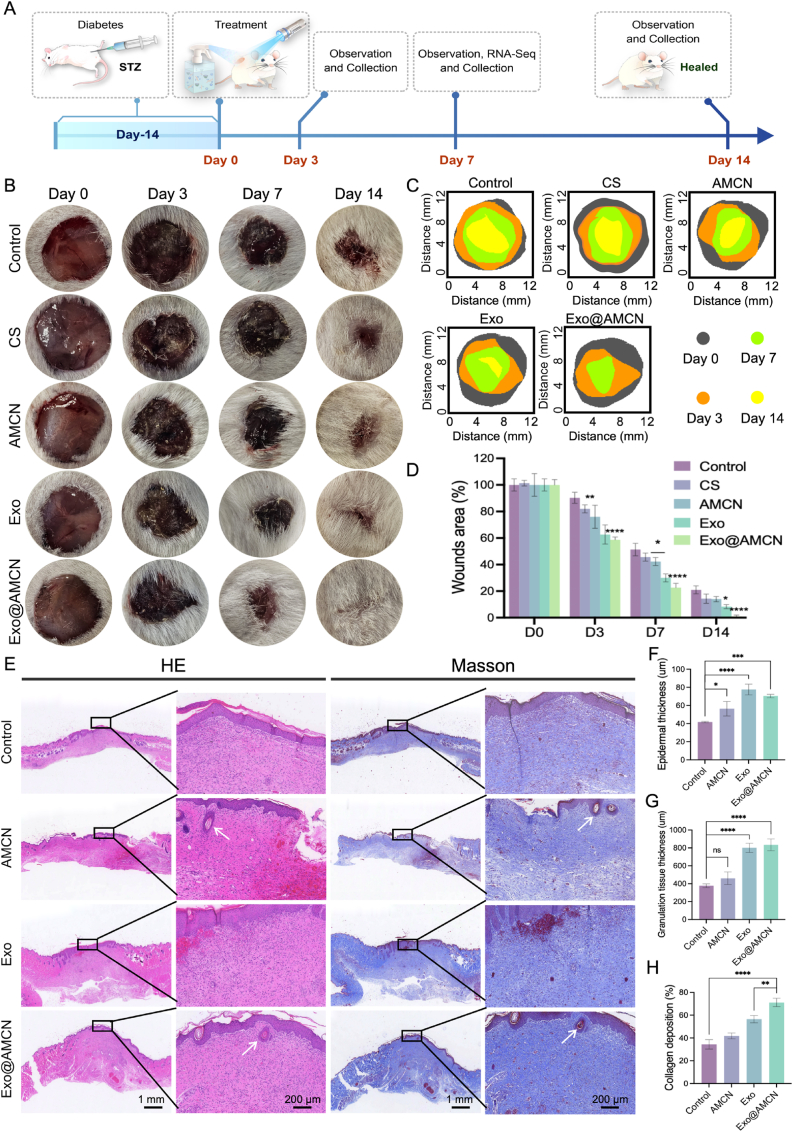
Hydrogel accelerates diabetic wound healing *in vivo*. (A) Schematic diagram and timeline of diabetic wound healing model establishment. (B) Representative optical images of wounds after implantation with different hydrogels. (C) Schematic illustration of wound closure and quantitative analysis. (D) Quantitative analysis of wound closure. (E) Representative H&E staining and Masson's trichrome staining images of wounds on day 14. (F) Analysis of epidermal thickness at wound sites. (G) Analysis of granulation tissue thickness at wound sites. (H) Quantitative analysis of collagen deposition percentages at wound sites. (∗*p* < 0.05, ∗∗*p* < 0.01, ∗∗∗*p* < 0.001, ∗∗∗∗*p* < 0.0001).

To evaluate the therapeutic efficacy of Exo@AMCN *in vivo*, full-thickness skin wounds were induced in diabetic mice (Fig. 5A). The wound healing process was monitored on postoperative days 0, 3,7, and 14 (Fig. 5B), with dynamic healing trajectories illustrated in Fig. 5C. All treatment groups exhibited progressive wound closure over time, with healing rates ranked as follows: Exo@AMCN > CS > Exo > AMCN > control (Fig. 5D). By day 7, Exo@AMCN (22.05 ± 4.17 %) and Exo (28.37 ± 4.39 %) groups showed significantly smaller wound areas compared to the control (51.49 ± 5.06 %) and AMCN (42.18 ± 2.86 %) groups (*p* < 0.05), confirming the pro-healing role of Exo on wound healing. Notably, AMCN outperformed the control group, suggesting that the ADM scaffold and borneol also contribute to diabetic wound repair. By day 14, Exo@AMCN achieved nearly complete wound closure (1.07 ± 1.27 %), surpassing the closure rates of CS (14.33 ± 3.51 %), AMCN (13.83 ± 1.90 %), Exo (8.25 ± 1.64 %), and control (21.04 ± 2.90 %) groups, highlighting the synergistic multi-mechanistic benefits of the composite hydrogel and giving it an advantage over commercial products.

Histopathological evaluation via H&E and Masson's trichrome staining (Fig. 5E) revealed enhanced epithelialization. By day 14, AMCN, Exo and Exo@AMCN groups exhibited significantly greater epithelial thickness than the control (*p* < 0.05). Particularly, Exo (77.63 ± 5.93 %) and Exo@AMCN (70.37 ± 1.92 %) showing the most pronounced effects (Fig. 5F), indicating the promotion of epidermal proliferation by both ADM and Exo. Besides, the thickness of granulation tissue, critical for supporting epithelial migration, was markedly increased in Exo@AMCN compared to the control group (*p* < 0.001) (Fig. 5G). Moreover, the Exo@AMCN group demonstrated significantly higher and more organized collagen deposition (*p* < 0.01) (Fig. 5H), indicating its potential to accelerate tissue regeneration and mitigate scar formation through enhanced extracellular matrix remodeling. Strikingly, neogenesis of hair follicles (arrowed in Fig. 5E) was observed in AMCN and Exo@AMCN groups, suggesting ADM-driven skin appendage regeneration for functional tissue restoration.

These results demonstrate that Exo@AMCN orchestrates multi-phase wound healing: accelerating granulation tissue formation initially and enhancing collagen remodeling in subsequent phase. The sustained release of Exo from Exo@AMCN activates the proliferation and migration of cells involved in the healing process, and its capacity to induce skin appendage regeneration may underpin accelerated functional tissue reconstruction.

### 3.6 Mechanisms of diabetic wound healing and biocompatibility assessment

**Fig. 6:**
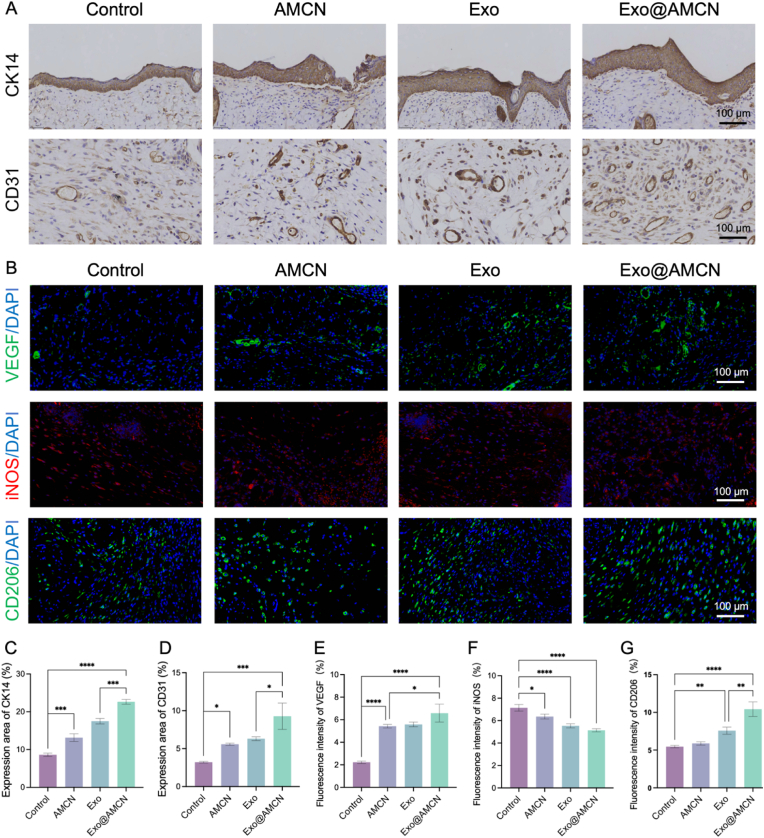
Immunohistological analysis of hydrogel-accelerated diabetic wound healing *in vivo*. (A) Representative images of CK14 and CD31 immunohistochemical staining. (B) Representative immunofluorescence images of VEGF, iNOS and CD206. (C) Quantitative analysis of CK14 expression area. (D) Quantitative analysis of CD31 expression area. (E) Quantitative analysis of VEGF fluorescence intensity. (F) Quantitative analysis of iNOS fluorescence intensity. (G) Quantitative analysis of CD206 fluorescence intensity. (∗*p* < 0.05, ∗∗*p* < 0.01, ∗∗∗*p* < 0.001, ∗∗∗∗*p* < 0.0001).

To investigate the mechanisms underlying Exo@AMCN's promotion of diabetic wound healing, immunohistochemistry was performed. CK14, a key marker of squamous epithelial regeneration, was used to assess re-epithelialization activity [44]. As shown in Fig. 6A, Exo@AMCN exhibited significantly higher CK14-positive staining intensity compared to other groups (*p* < 0.001) (Fig. 6C), indicating enhanced keratinocyte proliferation during re-epithelialization.

Angiogenesis, critical for tissue repair, was evaluated via CD31 labeling of vascular endothelial cells. Exo@AMCN demonstrated the largest CD31 positive area and most extensive vascular distribution among all groups (*p* < 0.05) (Fig. 6A and D). Furthermore, VEGF expression in AMCN, Exo, and Exo@AMCN groups was markedly elevated compared to the control (*p* < 0.05) (Fig. 6B and E), confirming that Exo@AMCN accelerates angiogenesis by upregulating VEGF, thereby shortening healing time.

The sustained pro-inflammatory microenvironment in diabetic wounds disrupts bioactive factor functionality and impedes healing [45]. Exo@AMCN, by inducing macrophage polarization, modulates inflammatory responses to improve the immune microenvironment. The fluorescence intensity of iNOS (M1 macrophage marker) followed the trend: control > AMCN > Exo > Exo@AMCN (Fig. 6F). Conversely, the fluorescence intensity of CD206 (M2 macrophage marker) was highest in Exo@AMCN, followed by Exo, and lowest in the control and AMCN groups (*p* < 0.01) (Fig. 6G). While early inflammation assists in debris clearance, excessive late-stage inflammation promotes fibrosis [46]. These results demonstrate that Exo@AMCN promotes M2 macrophage polarization, resolving chronic inflammation and enhancing diabetic wound repair.

In addition, histopathological analysis of major organs (heart, liver, spleen, lungs, kidneys) on postoperative day 7 revealed no abnormal pathological changes—such as inflammatory infiltration, fibrosis, or vacuolation—in any treatment group (Fig. S8), indicating preliminary biocompatibility. To comprehensively assess long-term biosafety, we extended observations to 28 days post-intervention. The experimental group exhibited complete wound closure with mature scar tissue formation, absent of adverse local reactions (e.g., mass formation or erythema) (Fig. S9). Crucially, systemic safety was corroborated by hematological and biochemical analyses: red blood cell (RBC), white blood cell (WBC) and liver/kidney function indices (AST,Cr) remained within normal physiological ranges (*p* > 0.05) (Fig. S11). Consistent with day 7 findings, histopathological re-examination of major organs at day 28 confirmed no pathological alterations (Fig. S10). Collectively, these data demonstrate that Exo@AMCN possesses robust biocompatibility and negligible systemic toxicity throughout the critical 28-day healing period, fulfilling essential translational safety criteria for diabetic wound therapeutics.

### 3.7 Transcriptomic analysis of wound healing acceleration mechanisms

**Fig. 7:**
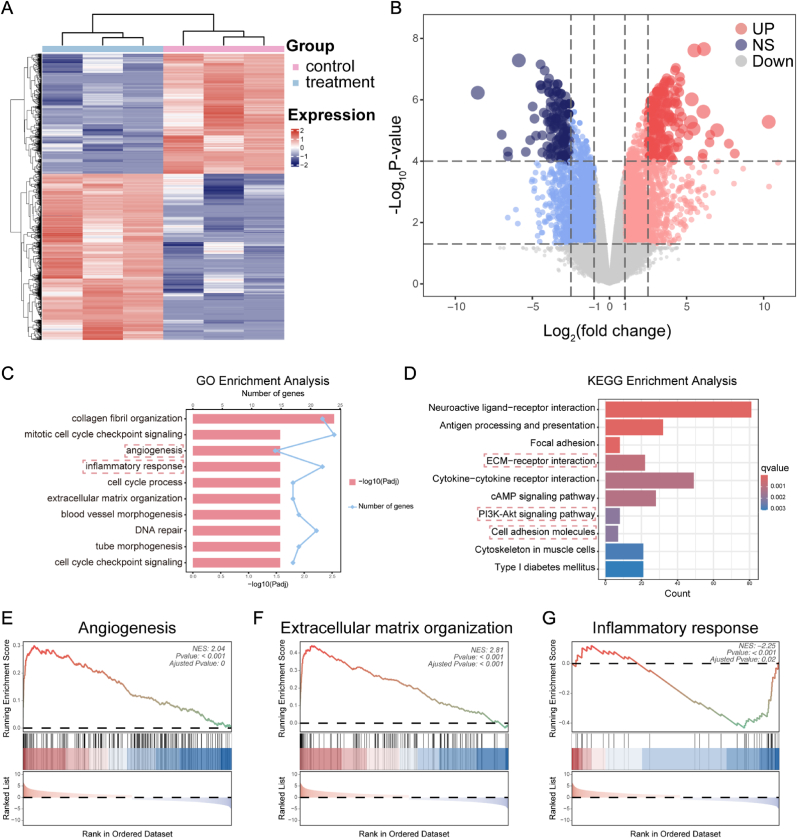
Transcriptomic analysis of potential mechanisms. (A) Heatmap of differentially expressed gene clustering. (B) Volcano plot of differentially expressed genes between groups. (C) GO enrichment analysis. (D) KEGG pathway enrichment analysis. (E) GSEA of angiogenesis pathway. (F) GSEA of extracellular matrix organization pathway. (G) GSEA of inflammatory response pathway.

To investigate the mechanisms underlying the promotion of diabetic wound repair by Exo@AMCN, transcriptomic sequencing was performed on wound tissues from control and Exo@AMCN-treated groups on postoperative day 7. Based on a fold change threshold of ≥2 (|log2FC| ≥1), 4426 differentially expressed genes (DEGs) were identified, including 2324 significantly upregulated and 2102 downregulated genes (Fig. 7A and B). Top 20 DEGs revealed pronounced upregulation of angiogenesis and proliferation-related genes (*SP7*, *Crhr1*, *Calhm4*, *Capn8)* and downregulation of inflammation-associated genes (*Serpina3h*, *H2-Ea*, *Nr1h4*, *Slc15a2*, *Hamp2*) (Supplementary Tables 1 and 2).

Gene Ontology (GO) analysis highlighted significant enrichment in biological processes (BP) including “angiogenesis” (GO:0001525), “inflammatory response” (GO:0006954), “ECM organization” (GO:0030,198), and “blood vessel morphogenesis” (GO:0048,514) (Fig. 7C). Kyoto Encyclopedia of Genes and Genomes (KEGG) pathway analysis further revealed activation of ECM-receptor interaction, PI3K/Akt signaling, and cell adhesion molecule-related pathways (Fig. 7D). Mechanistically, the ECM-receptor pathway promotes cell migration via integrin-mediated cell-matrix interactions, PI3K/Akt regulates proliferation and survival, and cell adhesion molecules (e.g., cadherins, selectins, integrins) mediate intercellular or cell-matrix adhesion [[47], [48], [49]].

Gene Set Enrichment Analysis (GSEA) corroborated these findings, showing significant activation of “angiogenesis” (NES = 2.024) and “ECM organization” (NES = 2.81) pathways (Fig. 7E and F), as well as a downregulation of NF-κB-mediated inflammatory responses (NES = −2.25) (Fig. 7G). These multi-omics insights demonstrate that Exo@AMCN accelerates diabetic wound healing through synergistic regulation of angiogenesis, inflammatory balance, ECM remodeling, and activation of key signaling networks (e.g., PI3K/Akt), forming a cascade of multi-pathway effect.

This mechanistic framework was further substantiated by qPCR validation of core regulatory genes. Specifically, significant upregulation of VEGF, PIK3CA, and AKT1 in Exo@AMCN-treated wounds functionally corroborates the activation of angiogenesis and PI3K/Akt signaling identified by KEGG/GSEA (Figs. S12 and 13). Concurrently, downregulation of TNF-α aligns with GSEA-inferred NF-κB pathway inhibition, attenuating pro-inflammatory that impede tissue repair (Fig. S12). These qPCR-validated gene dynamics demonstrate that Exo@AMCN achieves synergistic healing effects by PI3K/Akt pathway and NF-κB axis, thereby accelerating the transition from inflammatory to proliferative phase in diabetic wounds.

### 3.8 Potential mechanisms

In this study, we engineered a sprayable multifunctional hydrogel (Exo@AMCN) integrating synergistic antibacterial, ROS-scavenging, immunomodulatory, and pro-healing properties. The hydrogel was synthesized through methacrylated modification of ADM, enabling rapid photocrosslinking upon spraying onto wounds. *In vitro* and *in vivo* evaluations demonstrated its efficacy in accelerating diabetic wound healing. We hypothesize that the enhanced wound healing efficacy of Exo@AMCN may be attributed to the following mechanisms: (1) Natural borneol within Exo@AMCN imparts antibacterial effects in the initial stage of diabetic wound healing. Synergistically with Exo, it scavenges ROS, balances pro- and anti-inflammatory responses, and suppresses NF-κB-mediated inflammatory signaling, thereby shortening the inflammatory phase and maintaining tissue homeostasis. (2) During the proliferative phase, sustained release of Exo activates the PI3K-Akt pathway to promote fibroblast and keratinocyte proliferation, accelerating re-epithelialization. Concurrently, Exo upregulates VEGF expression, enhancing angiogenesis and granulation tissue formation for rapid wound closure. (3) The spatial topology and physicochemical properties of ADM in Exo@AMCN closely mimic native human ECM. This biomimetic scaffold delivers chemical and mechanical cues to direct cell adhesion, proliferation, and tissue regeneration via activation of ECM-receptor interaction pathways [47].

While Exo@AMCN demonstrates promising therapeutic potential, several limitations remain. Firstly, additional research is needed to clarify and confirm the molecular mechanisms responsible for its healing-promoting properties. Secondly, clinical applicability has not been verified in large animal models (e.g., porcine or non-human primates). Finally, the long-term metabolic fate of hydrogel degradation byproducts and potential immunogenicity remain unassessed. These limitations highlight critical directions for future research to advance the translational potential of this novel biomaterial.

## 4 Conclusion

In this study, we engineered a sprayable, exosome-loaded hydrogel (Exo@AMCN) with controlled therapeutic release, designed to address the multifactorial challenges of diabetic wound healing. This photocrosslinkable hydrogel, fabricated through methacrylation of decellularized dermal matrix, allows tailored therapeutic interventions by regulating the release of Exo and borneol to adapt to different stages of diabetic wound healing. *In vitro* models demonstrated the hydrogel's exceptional biocompatibility, antibacterial and antioxidant properties, ability to modulate inflammation, enhance keratinocyte migration, and stimulate angiogenesis. *In vivo* experiments demonstrated the superior wound healing efficacy of Exo@AMCN in comparison to AMCN hydrogel or Exo alone. Moreover, transcriptomics confirmed that Exo@AMCN promotes healing by upregulating angiogenesis- and inflammation-related genes in recipient cells, and activating pivotal signaling pathways like PI3K/Akt. This study offers a novel approach for the development of decellularized dermal matrix hydrogel. With its characteristics of adaptability and synergistic mechanism, this hydrogel has broad application prospects and great potential in the clinical transformation of treating diabetic wounds.

## CRediT authorship contribution statement

**Bo Liu:** Writing – original draft, Methodology, Investigation, Formal analysis, Data curation, Conceptualization. **Lianglong Chen:** Writing – original draft, Methodology, Investigation, Formal analysis, Data curation, Conceptualization. **Chaoyang Huang:** Writing – original draft, Investigation, Formal analysis, Data curation, Conceptualization. **HuiHui Zhang:** Methodology, Investigation. **Hai Zhou:** Methodology, Investigation. **Yanqi Chen:** Methodology, Investigation. **Xiaoyang Liu:** Project administration, Conceptualization. **Zhenyong Xiao:** Project administration, Conceptualization. **Kangyan Liang:** Project administration, Conceptualization. **Xiangtao Xie:** Writing – review & editing, Resources, Project administration, Funding acquisition. **Yi Gao:** Writing – review & editing, Resources, Project administration, Funding acquisition. **Kun Liu:** Writing – review & editing, Resources, Project administration, Funding acquisition. **Xiangdong Qi:** Writing – original draft, Resources, Project administration, Funding acquisition.

## Ethical statement

All procedures followed the guidelines for the care and use of laboratory animals and the ethical criteria outlined by the National Scientific Research Council.

## Declaration of competing interest

The authors declare that they have no known competing financial interests or personal relationships that could have appeared to influence the work reported in this paper.

## Data Availability

Data will be made available on request.
