# Supplementary material for: A sprayable exosome-loaded hydrogel with controlled release and multifunctional synergistic effects for diabetic wound healing
Source: Mater Today Bio. 2025 Aug 5;34:102159. doi: 10.1016/j.mtbio.2025.102159 (PMC12355498; doi:10.1016/j.mtbio.2025.102159)
Supplement: Multimedia component 1 [file mmc1.docx]

Supplementary Materials for

**Exosome-Loaded Acellular Dermal Matrix Hydrogel with Controlled Release and Multifunctional Synergy for Diabetic Wound Healing**

**Supplementary materials and methods**

Isolation and characterization of MSC-derived EVs

Isolation of MSC-derived EVs using ultracentrifugation: The supernatant was centrifuged at 3000×g for 15 min and 10000×g for 30 min at 4°C (CR21G, Hitachi, Japan) to remove dead cells and cell debris. Then, the supernatant was filtered through a 0.22 μm filter and centrifuged at 100,000×g at 4°C for 2 h (CP100NX, Hitachi, Japan). After centrifugation, the supernatant was carefully discarded, and the precipitate was resuspended in 1×PBS and centrifuged at 100,000×g at 4°C for 2 h. The bottom layer of liquid in the last centrifuge tube after discarding the supernatant contained the EVs. Furthermore, purified EVs were harvested by carefully pipetting this solution with 100 μl of PBS to completely dissolve the precipitate and stored at -80°C.

Nanoparticle tracking analysis (NTA): The EV particle size and concentration were measured by performing an NTA with a ZetaView PMX 110 instrument (Particle Metrix, Meerbusch, Germany) and the corresponding software ZetaView 8.04.02. The EVs were prediluted with 1×PBS and then injected into the sample carrier cell. The NTA measurements were recorded and analyzed at 11 positions. The ZetaView system was calibrated using 110 nm polystyrene particles. According to the EV amount detected using the NTA system, the amount of medium required to obtain the same amount of EVs was calculated.

Transmission electron microscopy (TEM): EV morphology was observed using a TEM (Tecnai G2 Spirit BioTWIN). The EV suspension was subsequently fixed with 2% PFA for 5 min, pipetted onto a carbon film, and incubated for 10 min. Then, the sample was negatively stained with 2% uranyl acetate (UA) and air-dried at RT. The EVs were observed by TEM for imaging.

Western blotting (WB): Western blotting was performed using standard protocols. EVs were harvested in 5 × radioimmunoprecipitation assay (RIPA) buffer, and endogenous proteins were extracted from cell lysates. Then, the protein concentration was determined with a BCA protein assay kit (Thermo Fisher Scientific). Samples with equivalent protein concentrations were separated on a 10% SDS-PAGE gel, transferred onto 0.22 μm polyvinylidene difluoride (PVDF) membranes (Millipore, MA), blocked with 5% fat-free milk and then incubated with primary antibodies overnight at 4°C. After washing with Tris-buffered saline-Tween (TBST) for 5 min, the membranes were incubated with secondary antibodies for 1 h. The protein bands were imaged with Clarity Western ECL Substrate (Bio-Rad, CA) and a VersaDoc 4000 MP (Bio-Rad). The following primary antibodies were used: anti-GAPDH (Abcam, ab8245), anti-CD63 (Abcam, ab134045), anti-Calnexin (Abcam, ab238078), and anti-TSG101 (BD Biosciences, BD612696).

Gene-specific primers：TNF-α: F 5'-CAGGCGGTGCCTATGTCTC-3',5'-CGATCACCCCGAAGTTCAGT-3'；IL-10: F 5'-GCTGGACAACATACTGCTAACCG-3', R 5'-GGCAACCCAAGGAGTGGTA-3'；VEGF: F 5'-CTACCTCCACCATGCCAAGT-3', R 5'-GCAGTAGCTGCGCTG-3'.

**Supplementary figures**

**
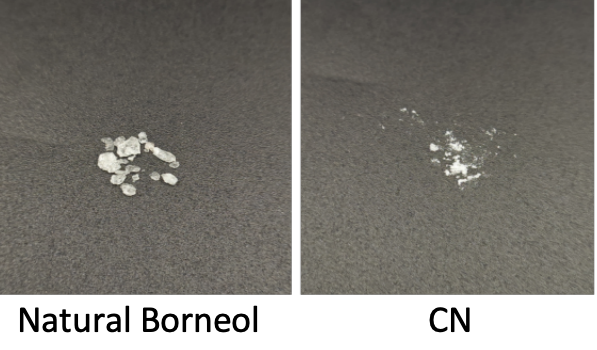
**

**Fig. S1.** Borneol transitioned from poorly soluble crystalline blocks to a soluble micropowder upon forming CN.


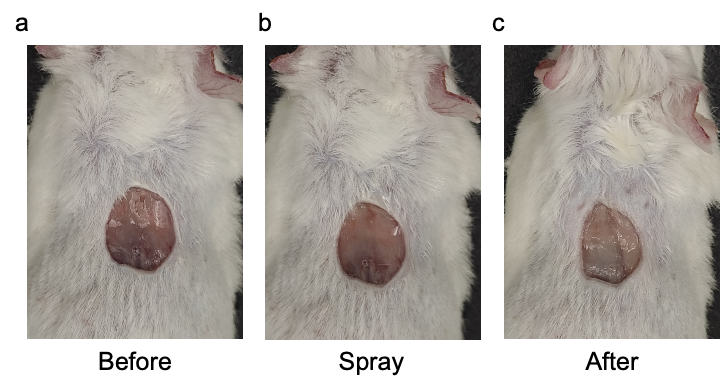


**Fig. S2.** Exo@AMCN applied to wound. a) A 10mm wound was made on the back of the mouse. b) Spray the hydrogel onto the wound surface. c) Gel is formed at the local wound after photocrosslinking.


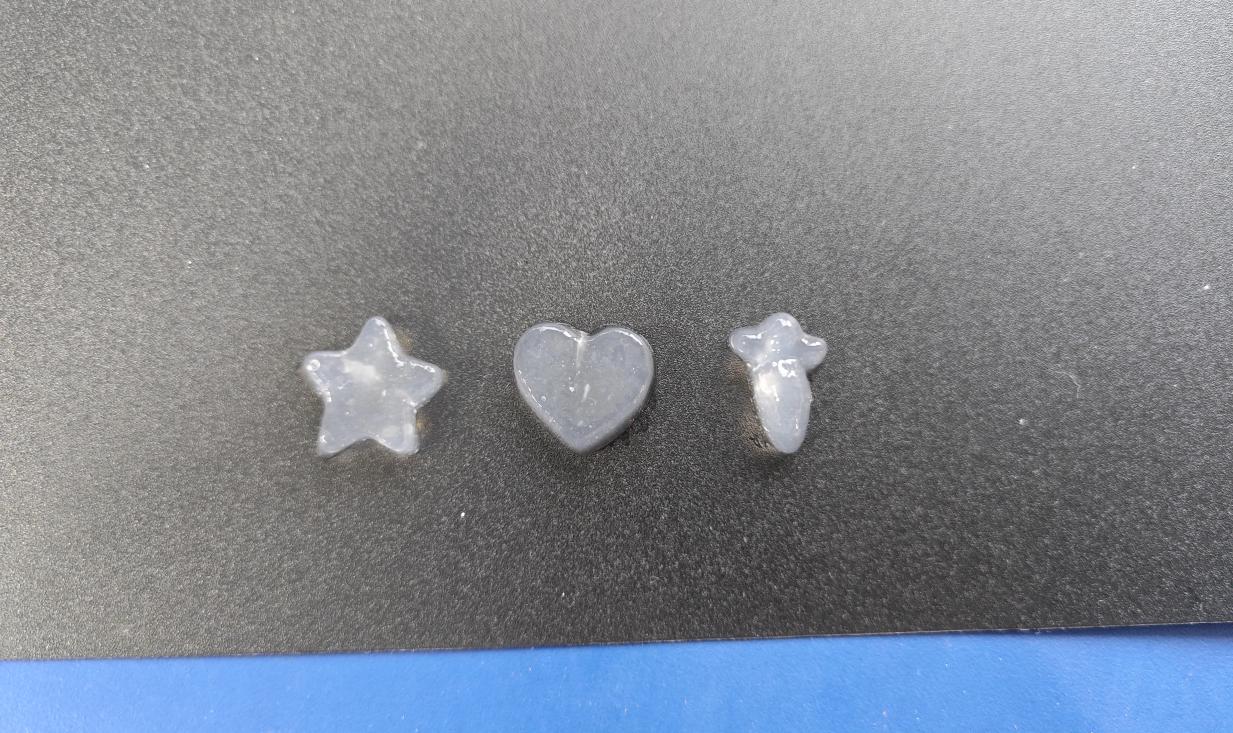


**Fig. S3.** Exo@AMCN formed uniform heart-shaped, star-shaped gels after cross-linking.


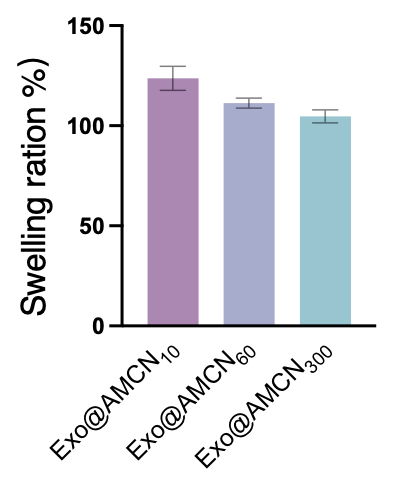


**Fig. S4.** Swelling ration of Exo@AMCN hydrogel


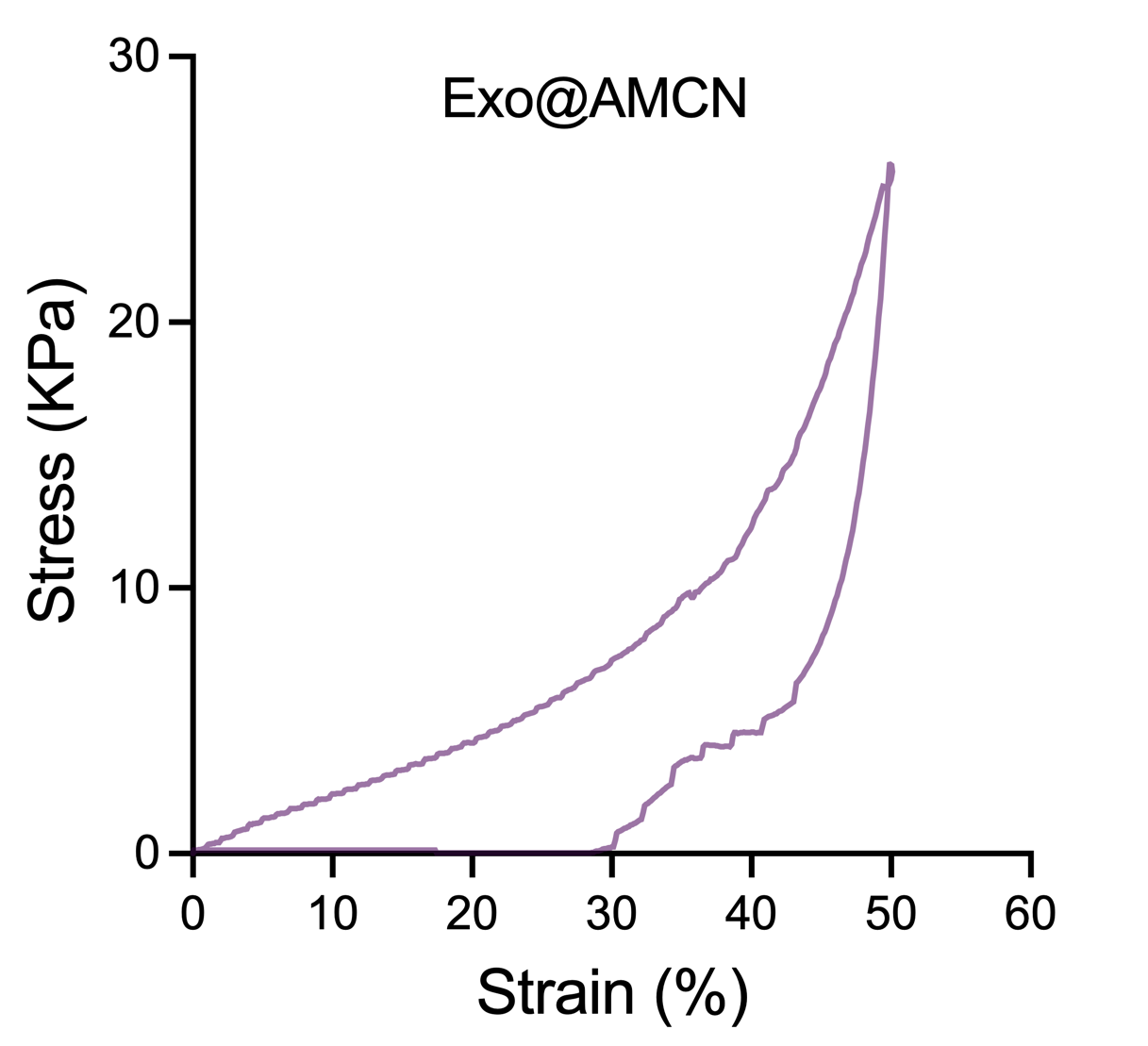


**Fig. S5.** Compressive stress-strain curve of Exo@AMCN hydrogel


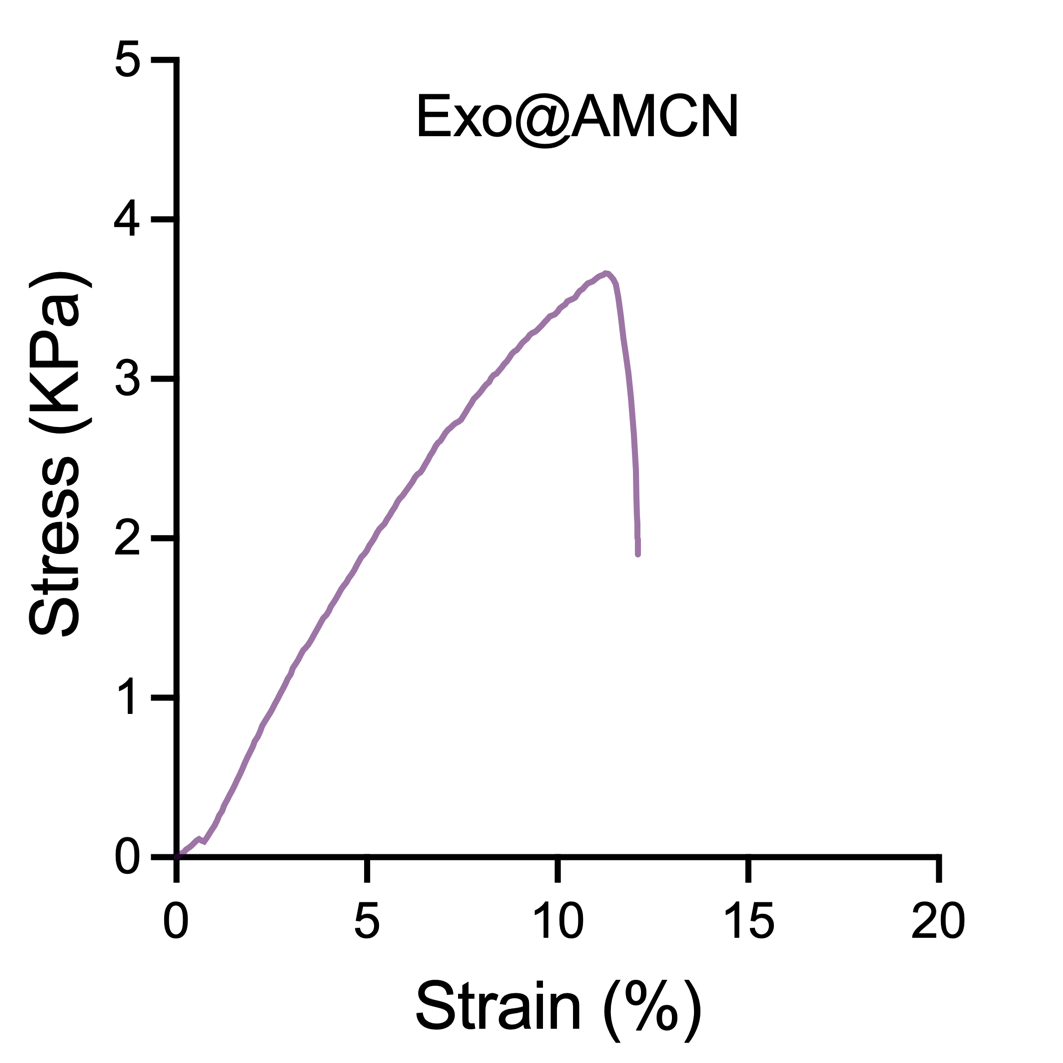


**Fig. S6.** Tensile stress-strain curve of Exo@AMCN hydrogel


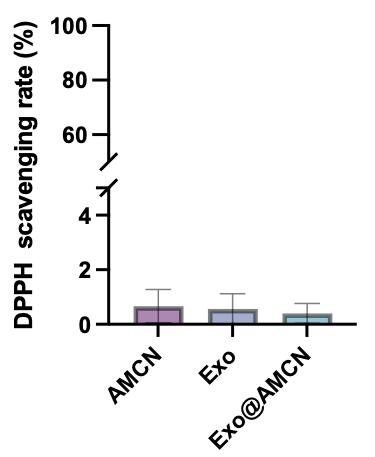


**Fig. S7.** DPPH scavenging assays.


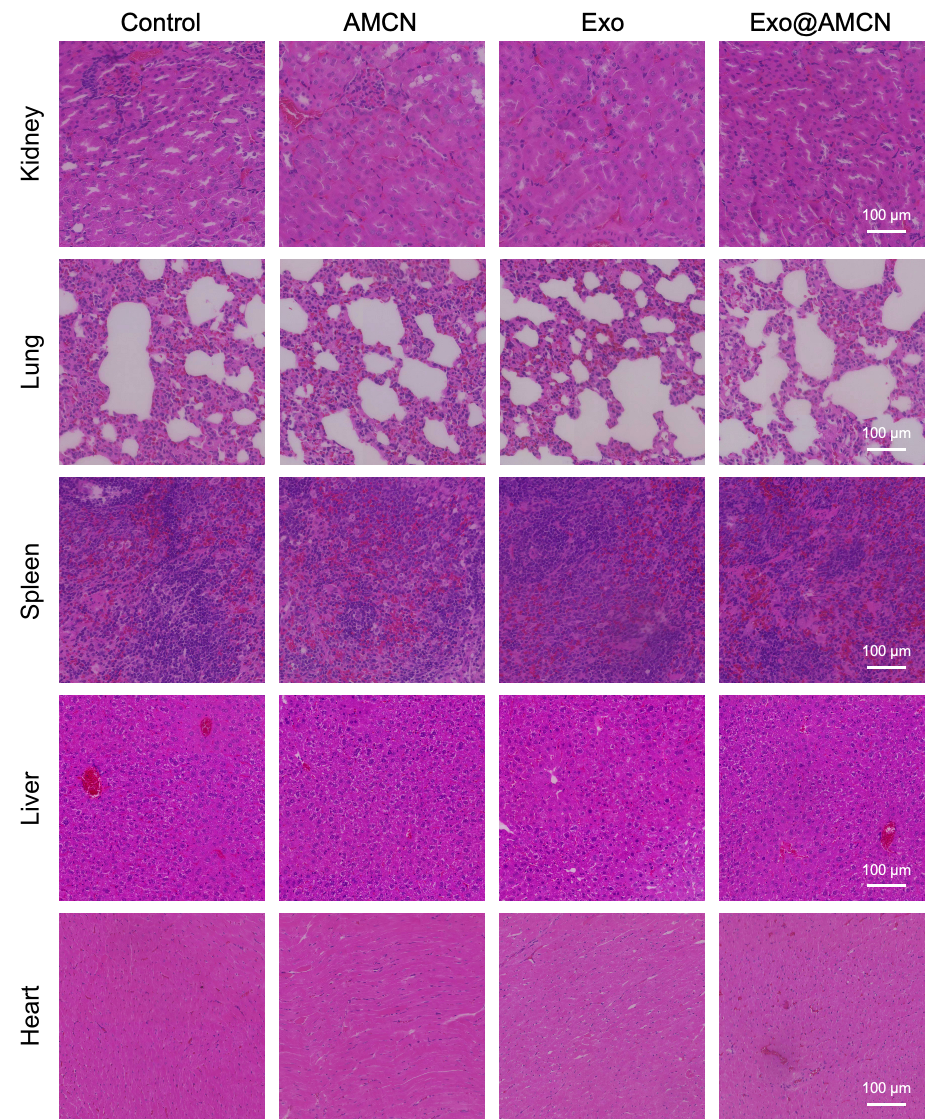


**Fig. S8.** H&E staining images of the heart, liver, spleen, lung, and kidney tissues on postoperative day 7.


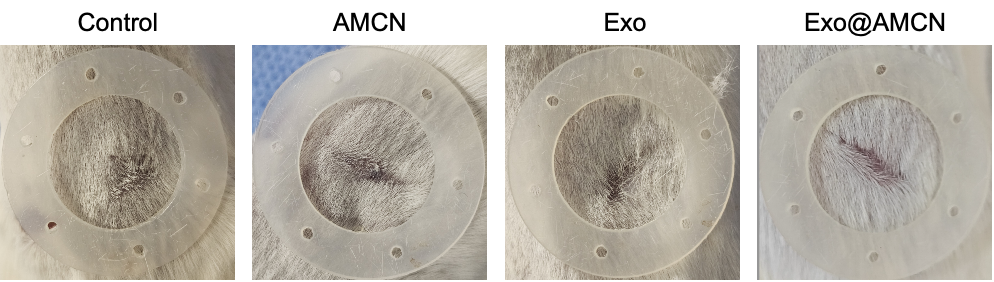


**Fig. S9.** Images of wounds on postoperative day 28.


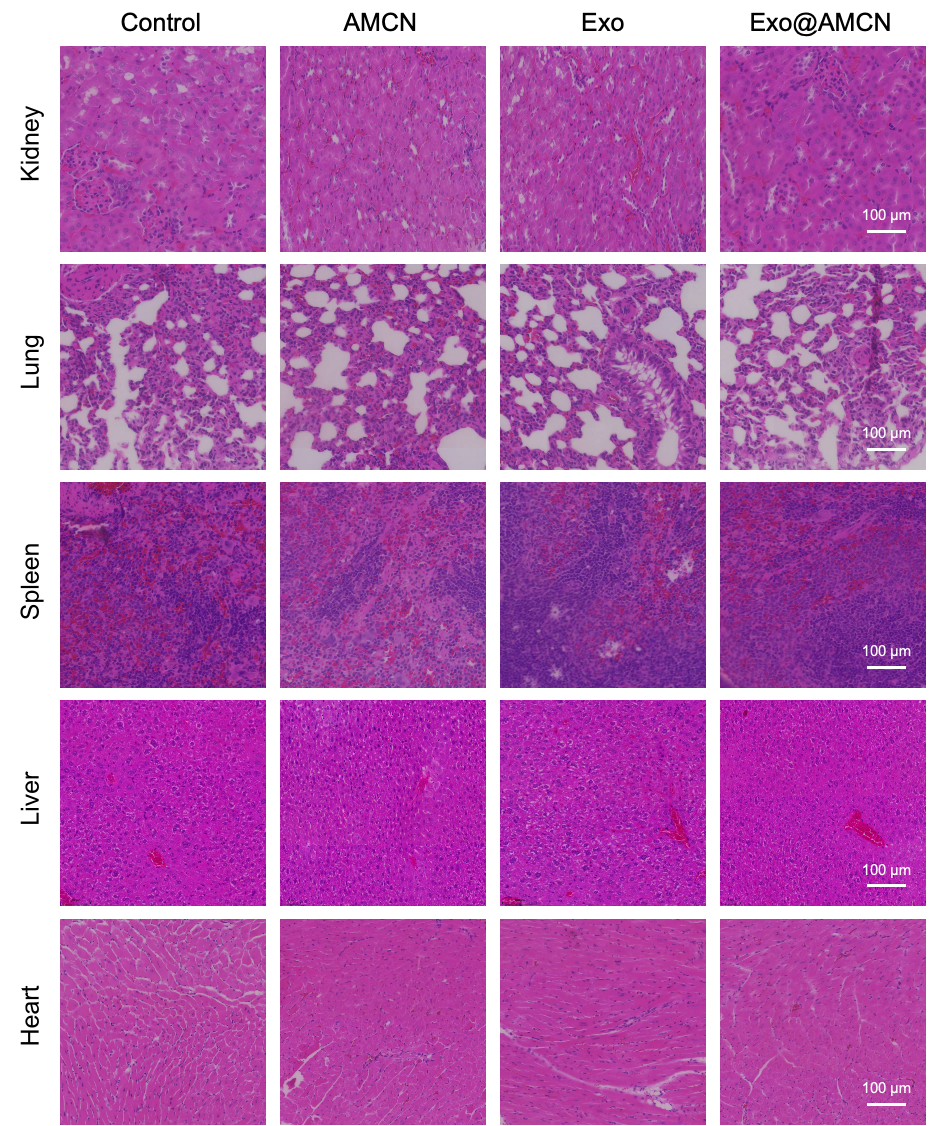


**Fig. S10.** H&E staining images of the heart, liver, spleen, lung, and kidney tissues on postoperative day 28.


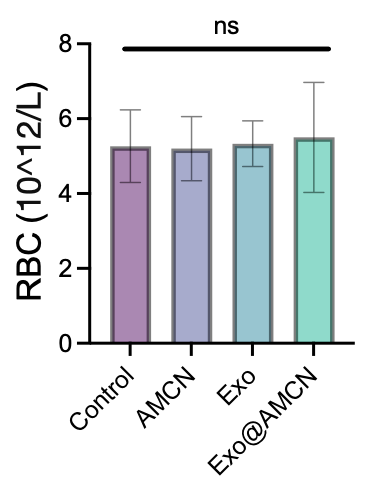

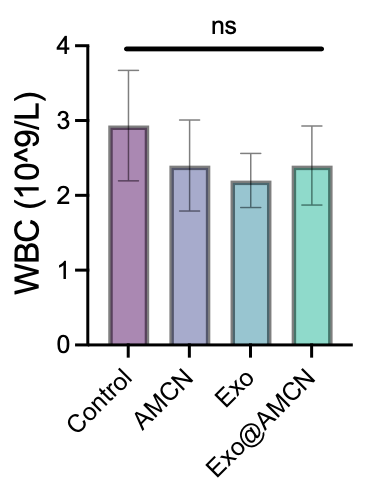


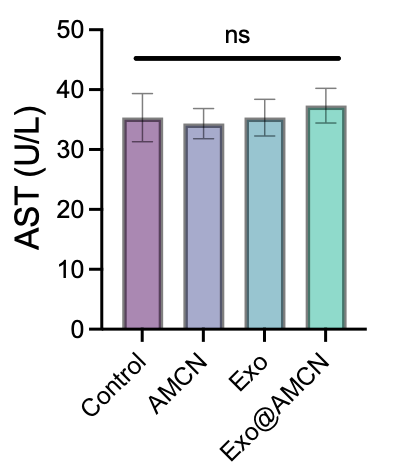

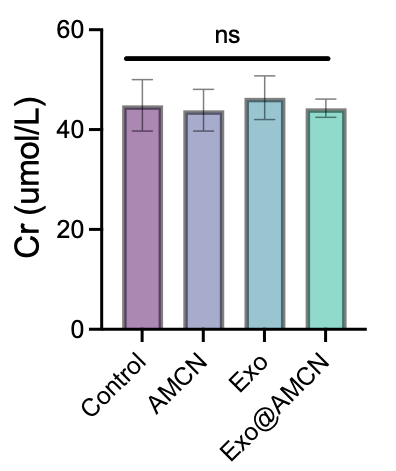


**Fig. S11.** Cell blood count and biochemical analysis on postoperative day 28.


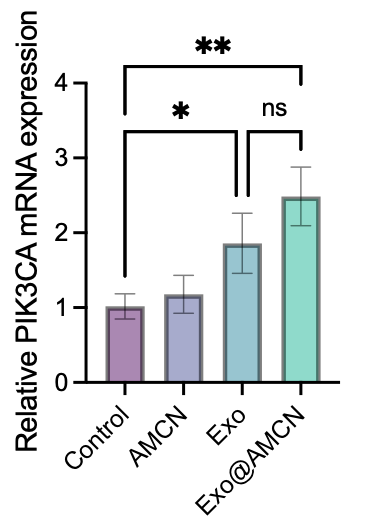

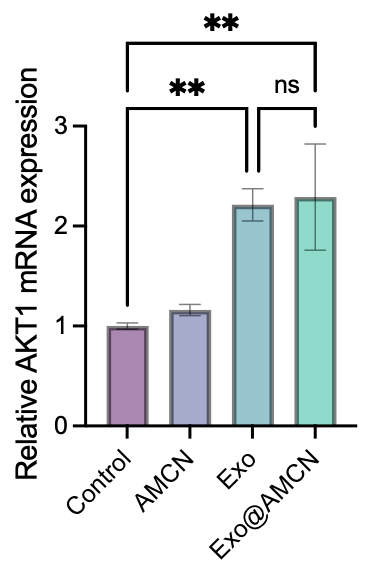


**Fig. S12.** Relative mRNA level of PIK3CA and AKT1 on the wound tissue.


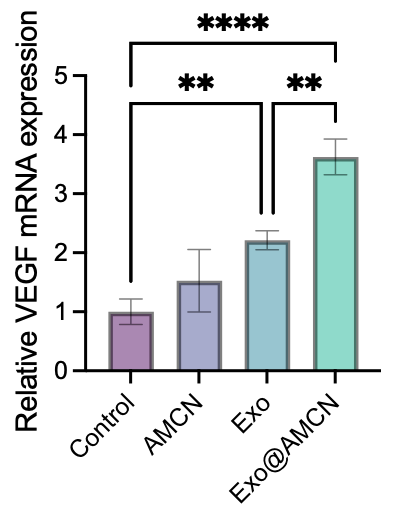

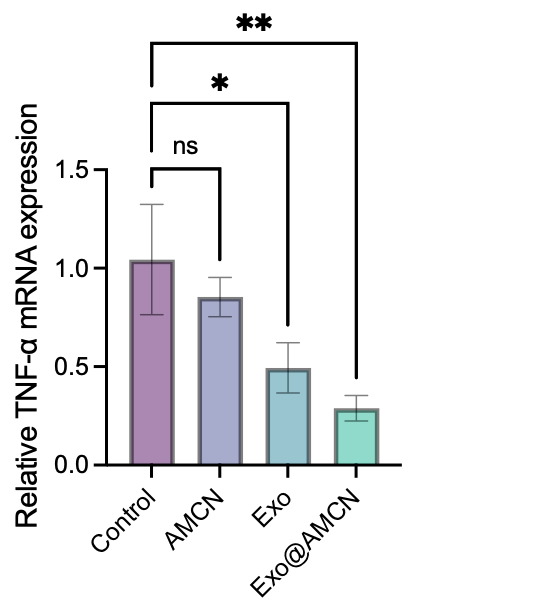


**Fig. S13.** Relative mRNA level of VEGF and TNF-α on the wound tissue.

**Supplementary tables**

**Table S1. The top 20 genes upregulate**

| **Gene** | **logFC** |
| --- | --- |
| Lyg2 | 8.6739 |
| Tgm6 | 8.1272 |
| Mup7 | 8.0785 |
| Krt35 | 7.7423 |
| Capn8 | 7.0580 |
| Kash5 | 6.9783 |
| Krt33b | 6.9318 |
| Gm36101 | 6.5336 |
| Krt73 | 6.4925 |
| Krt26 | 6.4558 |
| Gm49380 | 6.2604 |
| Calhm4 | 6.2254 |
| Gprc5d | 6.1569 |
| Crhr1 | 6.1251 |
| Sp7 | 6.0913 |
| Krt82 | 6.0898 |
| A030005K14Rik | 6.0853 |
| Krtap3-1 | 6.0620 |
| Krtap8-1 | 6.0305 |
| Mup12 | 5.9906 |

**Table S2. The top 20 genes downregulate**

| **Gene** | **logFC** |
| --- | --- |
| Serpina3h | -12.3471 |
| H2-Ea | -8.5479 |
| Gm2163 | -6.9919 |
| Gm28042 | -6.6020 |
| Gm49673 | -6.5926 |
| Inmt | -6.5832 |
| Krt18 | -6.0752 |
| Nr1h4 | -5.9533 |
| Gm22146 | -5.8840 |
| Slc15a2 | -5.4307 |
| ENSMUSG00000121829 | -5.1472 |
| Hamp2 | -5.0472 |
| Olig1 | -5.0113 |
| Gdf5 | -4.9853 |
| Gm48395 | -4.8921 |
| D630039A03Rik | -4.8673 |
| Fam3d | -4.8639 |
| Vwce | -4.8492 |
| Ces2c | -4.7965 |
| Gm16796 | -4.7621 |
